# Mitochondrial clearance of calcium facilitated by MICU2 controls insulin secretion

**DOI:** 10.1016/j.molmet.2021.101239

**Published:** 2021-04-28

**Authors:** N. Vishnu, A. Hamilton, A. Bagge, A. Wernersson, E. Cowan, H. Barnard, Y. Sancak, K.J. Kamer, P. Spégel, M. Fex, A. Tengholm, V.K. Mootha, D.G. Nicholls, H. Mulder

**Affiliations:** 1 Unit of Molecular Metabolism, Lund University Diabetes Center, Lund University, Malmö SE-205 02, Sweden; 2 Broad Institute of Harvard and MIT, Cambridge, MA 02142, USA; 3 Department of Medical Cell Biology, Uppsala University, Uppsala SE-751 23, Sweden; 4 Buck Institute for Research on Aging, Novato, CA 94945, USA

**Keywords:** Mitochondrial calcium uniporter, Voltage-dependent calcium channels, Bioenergetics, Knockout mice, Stimulus-secretion coupling

## Abstract

**Objective:**

Transport of Ca^2+^ into pancreatic β cell mitochondria facilitates nutrient-mediated insulin secretion. However, the underlying mechanism is unclear. Recent establishment of the molecular identity of the mitochondrial Ca^2+^ uniporter (MCU) and associated proteins allows modification of mitochondrial Ca^2+^ transport in intact cells. We examined the consequences of deficiency of the accessory protein MICU2 in rat and human insulin-secreting cells and mouse islets.

**Methods:**

siRNA silencing of *Micu2* in the INS-1 832/13 and EndoC-βH1 cell lines was performed; *Micu2*^−/−^ mice were also studied. Insulin secretion and mechanistic analyses utilizing live confocal imaging to assess mitochondrial function and intracellular Ca^2+^ dynamics were performed.

**Results:**

Silencing of *Micu2* abrogated GSIS in the INS-1 832/13 and EndoC-βH1 cells. The *Micu2*^−/−^ mice also displayed attenuated GSIS. Mitochondrial Ca^2+^ uptake declined in *MICU2*-deficient INS-1 832/13 and EndoC-βH1 cells in response to high glucose and high K^+^. *MICU2* silencing in INS-1 832/13 cells, presumably through its effects on mitochondrial Ca^2+^ uptake, perturbed mitochondrial function illustrated by absent mitochondrial membrane hyperpolarization and lowering of the ATP/ADP ratio in response to elevated glucose. Despite the loss of mitochondrial Ca^2+^ uptake, cytosolic Ca^2+^ was lower in si*MICU2*-treated INS-1 832/13 cells in response to high K^+^. It was hypothesized that Ca^2+^ accumulated in the submembrane compartment in *MICU2*-deficient cells, resulting in desensitization of voltage-dependent Ca^2+^ channels, lowering total cytosolic Ca^2+^. Upon high K^+^ stimulation, *MICU2*-silenced cells showed higher and prolonged increases in submembrane Ca^2+^ levels.

**Conclusions:**

MICU2 plays a critical role in β cell mitochondrial Ca^2+^ uptake. β cell mitochondria sequestered Ca^2+^ from the submembrane compartment, preventing desensitization of voltage-dependent Ca^2+^ channels and facilitating GSIS.

## Highlights

• MICU2 deficiency impairs glucose-stimulated insulin secretion.
• MICU2 deficiency abrogates mitochondrial calcium uptake.
• Depolarization-evoked cytosolic calcium increases are lower in *MICU2*-deficient cells.
• Mitochondria clear calcium from the subplasma membrane region, maintaining calcium influx.

## Graphical abstract

**Figure:**
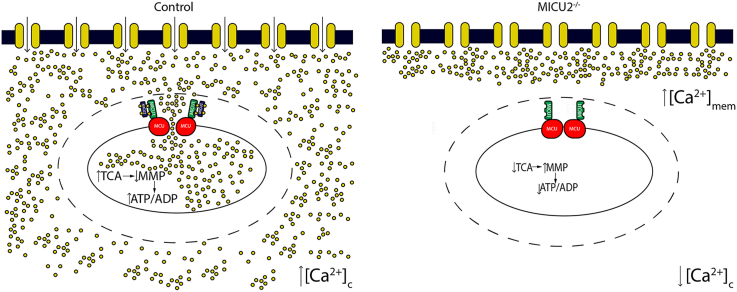

## 1 Introduction

While the canonical model of glucose-stimulated insulin secretion (GSIS) from pancreatic β cells with a central role of the mitochondrion has been established for more than 30 years, many details remain unclear. Extensive literature links mitochondrial Ca^2+^ uptake to facilitation of insulin secretion [[1], [2], [3], [4], [5], [6]]. However, it is unclear whether the reported effects of elevated matrix-free Ca^2+^ on insulin secretion are due to the bioenergetic consequences of the activation of citric acid cycle enzymes [7], the facilitation of plasma membrane oscillations [8], the generation of so-called coupling factors [5,9], or additional currently unrecognized mechanisms. The molecular identification of the components of the mitochondrial Ca^2+^ uniporter with the ability to selectively ablate one or more components allows the consequences of disrupted mitochondrial Ca^2+^ transport to be investigated in β cells.

The uniporter complex comprises the pore-forming subunit MCU plus up to six regulatory subunits [10]. MCU forms the complex's central pore-forming subunit, while MCU regulatory subunit b (MCUb), essential MCU regulator (EMRE), and mitochondrial uptake 1 and 2 (MICU1 and MICU2) form the accessory components [11]. As part of the Ca^2+^ uniporter holocomplex, MICU1 and MICU2 heterodimerize and act to maintain a threshold for mitochondrial Ca^2+^ uptake, upholding the mitochondrial Ca^2+^-buffering capacity [[12], [13], [14]]. It has been demonstrated in INS-1 832/13 insulinoma cells that MCU and MICU1 play a role in metabolic coupling; reduced expression of either decreases mitochondrial Ca^2+^ uptake and inhibits insulin secretion [8]. Furthermore, β cell-specific *Mcu* knockout mice display reduced first-phase insulin release in vivo and impaired mitochondrial Ca^2+^ uptake, glucose-induced ATP production, and insulin secretion in vitro [15]. However, the function of MICU2 in stimulus-secretion coupling in pancreatic β cells has not been examined. The situation in other cell types is somewhat unclear. In HeLa cells, silencing of *Micu2* leads to increased mitochondrial Ca^2+^ uptake [16,17], but only at cytosolic levels lower than 5 μM [17]. A similar phenotype is also observed in HEK-293T cells [13,18], while silencing of *Micu2* in mouse liver leads to impaired Ca^2+^ uptake kinetics [19]. To resolve the role of Ca^2+^ uptake, we examined the impact of MICU2 deficiency on insulin-secreting cells and mouse islets as well as in HEK-293T cells.

## 2 Materials and methods

### 2.1 Cell culture and model systems

INS-1 832/13 cells were cultured as previously described [20]. EndoC-βH1 cells [21] were grown on Matrigel-fibronectin-coated (100 μg/mL and 2 μg/mL, respectively, Sigma–Aldrich) culture vessels in DMEM containing 5.6 mM of glucose, 2% BSA fraction V (Roche Diagnostics), 10 mM of nicotinamide (Calbiochem), 50 μM of 2-mercaptoethanol, 5.5 μg/mL of transferrin, 6.7 ng/mL of sodium selenite (Sigma–Aldrich), 100 U/mL of penicillin, and 100 μg/mL of streptomycin (PAA). Both cell lines were cultured at 37 °C with 5% CO_2_. Unless otherwise stated, EndoC-βH1 cells were seeded at 2.3 × 10^5^ cells/cm^2^ and INS-1 832/13 cells at 1.5 × 10^5^ cells/cm^2^ in 24-well plates (Matrigel-fibronectin coated or uncoated) and transfected either with siRNA specific to *MICU2* (50 nM) or a scrambled siRNA (50 nM). Forty-eight h after knockdown, the growth media were changed to an overnight starvation medium containing 5.6 mM of glucose for INS-1 832/13 cells and 1 mM of glucose for EndoC-βH1 cells. HEK-293T cells were cultured in DMEM containing 1 g/L of d-glucose and supplemented with 10% heat-inactivated FBS, 100 U/mL of penicillin, and 100 μg/mL of streptomycin (PAA) [22]. The cells were plated at 1.5 × 10^5^ cells/cm^2^ in 24-well plates and transfected with either siRNA specific to *MICU2* or a scrambled siRNA. All of the assays were performed 48–72 h after knockdown.

### 2.2 *Micu2* knockout mice

We purchased a *Micu2* gene trap from the Texas A&M Institute of Genomic Medicine (College Station, TX, USA), bred the mice, and validated the disruption of the *Micu2* gene using standard procedures. The *Micu2* knockout mice had a B57BL/6J background and were extensively backcrossed. This mouse was previously characterized [23]. A combination of male and female mice was used with no significant differences between the two sexes.

### 2.3 Insulin secretion assay

INS-1 832/13 cells were incubated in HEPES-balanced salt solution (HBSS; 115 mM of NaCl, 5 mM of KCl, 1 mM of MgSO_4_, 1 mM of CaCl_2_, 25.5 mM of NaHCO_3_, 20 mM of HEPES, and 0.2% BSA at a pH of 7.2) containing 2.8 mM of glucose for 2 h followed by 1 h of stimulation with HBSS containing 2.8 or 16.7 mM of glucose or 15 min of stimulation with HBSS containing 36 mM of KCl and 2.8 mM of glucose [20]. Insulin secretion was measured with a rat insulin ELISA kit (Mercodia A/B, Sweden) according to the manufacturer's instructions. EndoC-βH1 cells were starved overnight in 1 mM of glucose-containing growth medium 1 day before the experiment. On the day of the experiment, the growth medium was removed and replaced with 1 mM of glucose-containing HBSS for 2 h followed by 1 h of stimulation with HBSS containing 1 or 20 mM of glucose [24]. Insulin secretion was measured with a human insulin ELISA (Mercodia A/B, Sweden) according to the manufacturer's instructions.

### 2.4 RNA isolation and quantitative real-time PCR

Total RNA was extracted from EndoC-βH1 cells, INS-1 832/13, and HEK-293T cells using an RNeasy Mini kit (Qiagen) according to the manufacturer's protocol. RNA concentrations were determined using a NanoDrop Spectrophotometer (Thermo Fisher Scientific). Then 1000 ng of total RNA were reverse transcribed using a RevertAid First-Strand cDNA synthesis kit (Fermentas, Vilnius, Lithuania). Quantitative real-time PCR (qPCR) was performed using TaqMan gene expression assays (*EFHA1/Micu2* rat: s235193; *EFHA1/Micu2* human: s47976; *Hprt1* rat: Rn01527840; *Hprt1* human*:* Hs99999909; *Mcu* rat: Rn01433739; *Micu1* rat: Rn01644475; *Cacna1a* rat, Rn00563825; *Cacna1c* rat, Rn00709287; and *INS1* mouse, Mm01950294, Applied Biosystems, Life Technologies, Carlsbad, CA, USA) using a 7900HT Fast Real-Time System (Applied Biosystems). qPCR was carried out as follows. Five ng of cDNA were mixed with 0.5 μL of gene assay and 5 μL of TaqMan Master Mix and ddH_2_O to a final volume of 10 μL and qPCR was performed using the following program: 50 °C for 2 min, 95 °C for 10 min, 40 cycles of 95 °C for 15 s, and 60 °C for 1 min. In all of the runs, a 10x dilution series was produced of one of the samples and used to generate a calibration curve as well as quality control for amplification efficiency. Gene expression was relatively quantified using the calibration curve. The amount of mRNA was calculated relative to the amount of hypoxanthine-guanine phosphoribosyl transferase (*HPRT1*) as a reference gene.

### 2.5 SDS-PAGE and western blotting

Cells were harvested in RIPA buffer followed by lysis by shaking the samples for 30 min at 950 rpm and 4 °C for 30 min. This was followed by centrifugation at 16,000*g* and 4 °C for 20 min to remove cell debris. Supernatants/lysates were kept at −80 °C until use. Then 10–20 μg of protein were separated on mini-PROTEAN TGX pre-stained gels (Bio-Rad) followed by activation of pre-stained Bio-Rad MP imager. The proteins were blotted onto Trans-Blot Turbo Transfer Packs (Bio-Rad), using Bio-Rad semidry blotter, and total protein for loading control was determined from the pre-stains. Membranes were blocked with 10% BSA or 5% skim milk (for mitochondrial complexes I–V experiments) in TRIS-buffered saline (TBS) unless otherwise stated and hybridized with antibodies for MICU2/EFHA1 (Abcam, ab101465), β-tubulin (Abcam, ab6046), α-tubulin (Abcam, ab7291), and mitochondrial complexes I–V (Abcam, ab110413).

### 2.6 Single-cell live cytosolic ATP and ATP/ADP ratio measurements using perceval and perceval HR

Single-cell cytosolic ATP/ADP ratio measurements were carried out in INS-1 832/13 cells using the genetically encoded biosensor Perceval HR [25]. The cells were seeded on poly-d-lysine-coated 8-well-chambered cover glasses (Lab-Tek, Thermo Fisher Scientific) at a density of 70,000 cells/cm^2^. Twenty-four h after seeding, the cells were co-transfected with siRNA and 1 μg of plasmid-encoding Perceval HR (Addgene ID: 49,083) at approximately 50% cell confluency. The cells were further grown for 48–72 h before measurements. On the day of imaging, the cells were pre-incubated at 37 °C in 400 μL of buffer P (135 mM of NaCl, 3.6 mM of KCl, 1.5 mM of CaCl_2_, 0.5 mM of MgSO_4_, 0.5 mM of Na_2_HPO_4_, 10 mM of HEPES, and 5 mM of NaHCO_3_ at a pH of 7.4) containing 2.8 mM glucose. After 1.5 h of incubation, the cells were imaged at 488 nm excitation and 505–535 nm emission recorded on a Zeiss LSM510 inverted confocal fluorescence microscope. Mouse islets were transduced with adenovirus expressing the similar ATP biosensor Perceval [26,27]. Poly-d-lysine-coated 8-well-chambered cover glasses (Lab-Tek, Thermo Fisher Scientific) containing mouse islets were incubated for 2 h with RPMI with 10% FBS, 100 U of penicillin, and 100 mg/mL of streptomycin containing the Perceval adenovirus. Then this medium was replaced with fresh RPMI medium with 10% FBS, 100 U of penicillin, and 100 mg/mL of streptomycin and incubated overnight. On the day of imaging, the cells were pre-incubated at 37 °C in 400 μL of buffer P (135 mM of NaCl, 3.6 mM of KCl, 1.5 mM of CaCl_2_, 0.5 mM of MgSO_4_, 0.5 mM of Na_2_HPO_4_, 10 mM of HEPES, and 5 mM of NaHCO_3_ at a pH of 7.4) containing 2.8 mM of glucose. After 1.5 h of incubation, the cells were imaged as described for clonal cell lines at 488 nm excitation and emission recorded at 505–530 nm on a Zeiss LSM510 inverted confocal fluorescence microscope using a 40x/0.75 objective. Images were recorded at a frequency of 0.25 Hz (scan time = 3.93 s). Equimolarity was accounted for in the 36 mM KCl solution.

### 2.7 Single-cell live cytoplasmic-free Ca^2+^ measurements

Cells were seeded onto poly-d-lysine coated 8-well-chambered cover glasses (Lab-Tek, Naperville, IL, USA) at a density of 70,000 cells/cm^2^ per well. Twenty-four h after seeding, the cells were transfected with siRNA and incubated for further 48–72 h prior to imaging. On the day of imaging, the cells were pre-incubated at 37 °C in 400 μL of buffer P containing 2.8 mM of glucose. After 1.5 h of incubation, based on variable Ca^2+^ affinities, INS-1 832/13 and HEK-293T cells were loaded with 2 μM of Fluo4 AM and Fluo5F AM, respectively. Incubation was continued for an additional 30 min. Immediately prior to imaging, the pre-incubation buffer was exchanged with 400 μL of buffer P. Fluo4 AM and Fluo5F AM were excited at 488 nm and emission recorded using a 505 nm emission filter on a Zeiss LSM510 inverted confocal fluorescence microscope using a 40x/0.75 objective. Images were recorded at a frequency of 0.64 Hz (scan time = 1.57 s) using a pinhole diameter of 463 μm. Single-cell-free cytoplasmic Ca^2+^ traces were displayed in arbitrary fluorescent units.

For experiments performed with the line scan configuration, INS-1 832/13 cells and EndoC-βH1 cells were plated, transfected, and loaded as previously described, except Fluo8 AM (2 μM) used instead in EndoC-βH1 cells. Imaging was again performed using a Zeiss LSM510 inverted confocal fluorescence microscope with a 100x/1.45 objective with Fluo4/Fluo8 excited at 488 nm and emission recorded using a 505 nm emission filter. For each recording, a single line was drawn through the center of 1–3 cells. This was the only part of the field of view that was measured during the recording. The pinhole diameter was reduced to 256 μm to minimize signals from outside the focal plane (1.4 μm section). This allowed the Ca^2+^ signals to be tracked as they propagated from the edge to the center of the cells with minimal interference from Ca^2+^ diffusing from above or below the focal plane. Recordings were performed at a frequency of 333 Hz (scan time = 3 ms) and a duration of 6000 cycles.

### 2.8 Single-cell live mitochondrial Ca^2+^ measurements

Single-cell [Ca^2+^]_mito_ measurements were carried out using genetically encoded mitochondrial-targeted Ca^2+^ biosensor mito-case12 (Evrogen cat# FP992.). INS-1 832/13 and HEK-293T cells were seeded onto poly-d-lysine-coated 8-well-chambered cover glasses (Lab-Tek, Thermo Fisher Scientific) at a cell density of 70,000 cells/cm^2^. Twenty-four h after seeding, the cells were co-transfected with siRNA and 1 μg of plasmid encoding mito-case12 at approximately 50% cell confluency. The cells were further grown for 48–72 h before measurements. On the day of imaging, the cells were pre-incubated at 37 °C in 400 μL of buffer P containing 2.8 mM of glucose. After 1.5 h, the cells were imaged using a 488 nm laser and emission recorded at 505–530 nm on a Zeiss LSM510 inverted confocal fluorescence microscope using a 100x/1.45 objective. Images were recorded at a frequency of 0.25 Hz (scan time = 3.93 s).

### 2.9 Live cell mitochondrial-free Ca^2+^ measurements using Rhod2 AM

EndoC-βH1 cells were seeded onto poly-d-lysine-coated 8-well-chambered cover glasses at a density of 70,000 cells/cm^2^ (Lab-Tek, Naperville, IL, USA). The day after seeding, the cells were transfected with siRNA and incubated for 72 h prior to imaging. On the day of imaging, the cells were pre-incubated at 37 °C in 400 μL of buffer P containing 2.8 mM of glucose. After 1.5 h of incubation, 2 μM of Rhod-2 AM (Abcam) was loaded for 30 min at 37 °C in buffer P. The cells were then washed once with buffer P to remove excess dye prior to imaging. Excitation of Rhod-2 AM was performed at 543 nm and emission recorded at 590–615 nm on a Zeiss LSM510 inverted confocal fluorescence microscope. For single mouse pancreatic islet mitochondrial-free Ca^2+^ measurements, poly-d-lysine-coated 8-well-chambered cover glasses containing single complete or partially digested mouse pancreatic islets pre-incubated at 37 °C in 400 μL of buffer P containing 2.8 mM of glucose. After 1.5 h of incubation, 2 μM of Rhod-2 AM (Abcam) was loaded for 30 min at 37 °C in buffer P. The cells were then washed once with buffer P to remove excess dye prior to imaging. Excitation of Rhod-2 AM was performed at 543 nm and emission recorded at 565–615 nm. The examination of the effect of glucose on [Ca^2+^]_mito_ was performed on a Zeiss LSM510 inverted confocal fluorescence microscope using a 100x/1.45 objective. Images were recorded at a frequency of 0.13 Hz (scan time = 7.86 s).

### 2.10 Simultaneous measurement of cell cytoplasmic-free Ca^2+^ and cell mitochondrial-free Ca^2+^

For experiments measuring the effect of 36 mM of KCl on [Ca^2+^]_c_ and [Ca^2+^]_mito_ in EndoC- βH1, the protocol was the same except that after 1.5 h of incubation, 2 μM of Rhod-2 AM (Abcam) and 2 μM of Fluo8 AM were loaded for 30 min at 37 °C in buffer P. Imaging was performed on a Zeiss LSM510 inverted confocal fluorescence microscope using a 40x/0.75 objective with images recorded at a frequency of 0.30 Hz (scan time = 3.36 s).

### 2.11 Single-cell live mitochondrial membrane potential (Δψ_m_) measurements

INS-1 832/13 cells were seeded onto poly-d-lysine-coated 8-well-chambered cover glasses (Lab-Tek, Naperville, IL, USA) at a density of 70,000 cells/cm^2^. Twenty-four h after seeding, the cells were transfected with siRNA and incubated for 48–72 h prior to imaging. For Δψ_m_ measurements, the cells were pre-incubated for 2 h in buffer P with 100 nM of TMRM (Invitrogen) and 2.5 μM of cyclosporin A. After incubation, the cells were washed once with buffer P and then imaging was performed. Under these conditions, Δψ_m_ measurements were performed in quench mode [28]. TMRM was present in the medium throughout the experiment. TMRM fluorescence measurements were performed using 543 nm excitation and a 560 nm long pass emission filter on a Zeiss LSM510 inverted confocal fluorescence microscope using a 40x/0.75 objective. Images were recorded at a frequency of 0.64 Hz (scan time = 1.57 s).

### 2.12 Single-cell live Subplasmalemmal Ca^2+^ measurements

Single-cell [Ca^2+^]_mem_ measurements were carried out using PeriCam-based genetically encoded inner plasma membrane-targeted Ca^2+^ probe mem-case12 (Evrogen cat# FP992.). Specifically, INS-1 832/13 and HEK-293T cells were seeded onto poly-d-lysine-coated 8-well-chambered cover glasses (Lab-Tek, Naperville, IL, USA) at a cell density of 70,000 cells/cm^2^. After 24 h of seeding, the cells were co-transfected with siRNA and 1 μg of plasmid-encoding mem-case12 at approximately 50% cell confluency. The cells were then further grown for 48–72 h before measurements. On the day of imaging, the cells were pre-incubated at 37 °C in 400 μL of buffer P containing 2.8 mM of glucose. After 1.5 h, the cells were imaged at 488 nm excitation (8%) and emission recorded at 505–535 nm on a Zeiss LSM510 inverted confocal fluorescence microscope using a 100x/1.45 objective. Images were recorded at a frequency of 0.64 Hz (scan time = 1.57 s).

### 2.13 Mouse islet insulin secretion and insulin content

Islets were picked by hand as previously described [29] under a stereomicroscope and incubated in HBSS containing 2.8 mM of glucose for 2 h. This was followed by 1 h of stimulation with HBSS supplemented with 2.8 or 16.7 mM of glucose. Aliquots of incubation medium were collected and insulin secretion was measured with a mouse insulin ELISA kit (Mercodia A/B, Sweden) according to the manufacturer's instructions. To extract insulin from mouse islets for insulin content measurements, 300 μl of acid ethanol (95% ethanol, HCL (37%), and water) were added to tubes containing 10 islets. The tubes were then sonicated 3 times for 5 s. The samples then underwent 5 cycles of freezing/thawing consisting of 5 min of freezing in ethanol/dry ice followed by 5 min of thawing on ice. The samples were the centrifuged for 5 min at 13.4×*g* with 50 μl of supernatant removed into a new tube, stored at −20 °C, and then measured using mouse insulin ELISA.

### 2.14 Glucose and insulin tolerance tests

All tolerance tests were performed in anesthetized mice, using 0.25 mg of midazolam (Midazolam Panpharma, Fougères, France) combined with 0.5 mg of fluanisone and 0.02 mg of fentanyl (Hypnorm, VetPharma, Leeds, UK). For IVGTT, mice were starved for 2 h prior to tail vein injection with 1 g/kg of d-glucose. For ITT, unfasted mice were intraperitoneally injected with 0.75 mU/g of human insulin (Novo Nordisk, Clayton, NC, USA). In each case, blood was collected via retro-orbital bleeding at the indicated time points after injection. Upon completion, the samples were centrifuged and plasma collected and stored at −20 °C. Insulin and glucose concentrations were then determined by a mouse insulin ELISA kit (Mercodia, Uppsala, Sweden) and Infinity Glucose (Oxidase) Reagent (Thermo Fisher Scientific, Waltham, MA, USA), respectively. Glucose measurements for ITT were determined by an Amplex Red Glucose/Glucose Oxidase Assay kit (Thermo Fisher Scientific, Waltham, MA, USA).

### 2.15 Statistical analyses

All of the experiments were repeated on 3 different days with the exception of ITT, which was performed twice. Data in all of the figures are presented as mean ± SEM, and unless specifically stated; statistical significance was determined by unpaired two-tailed Student's t-tests. For imaging experiments, n in the figures represents the number of cells or regions of interest (ROIs). For islet insulin secretion and IVGT assays, n in the figures represents the number of animals. For all of the statistical comparisons, a *p* value of less than 0.05 was considered significant.

## 3 Results

### 3.1 Functional consequences of MICU2 knockdown in insulin-secreting cells

**Figure 1:**
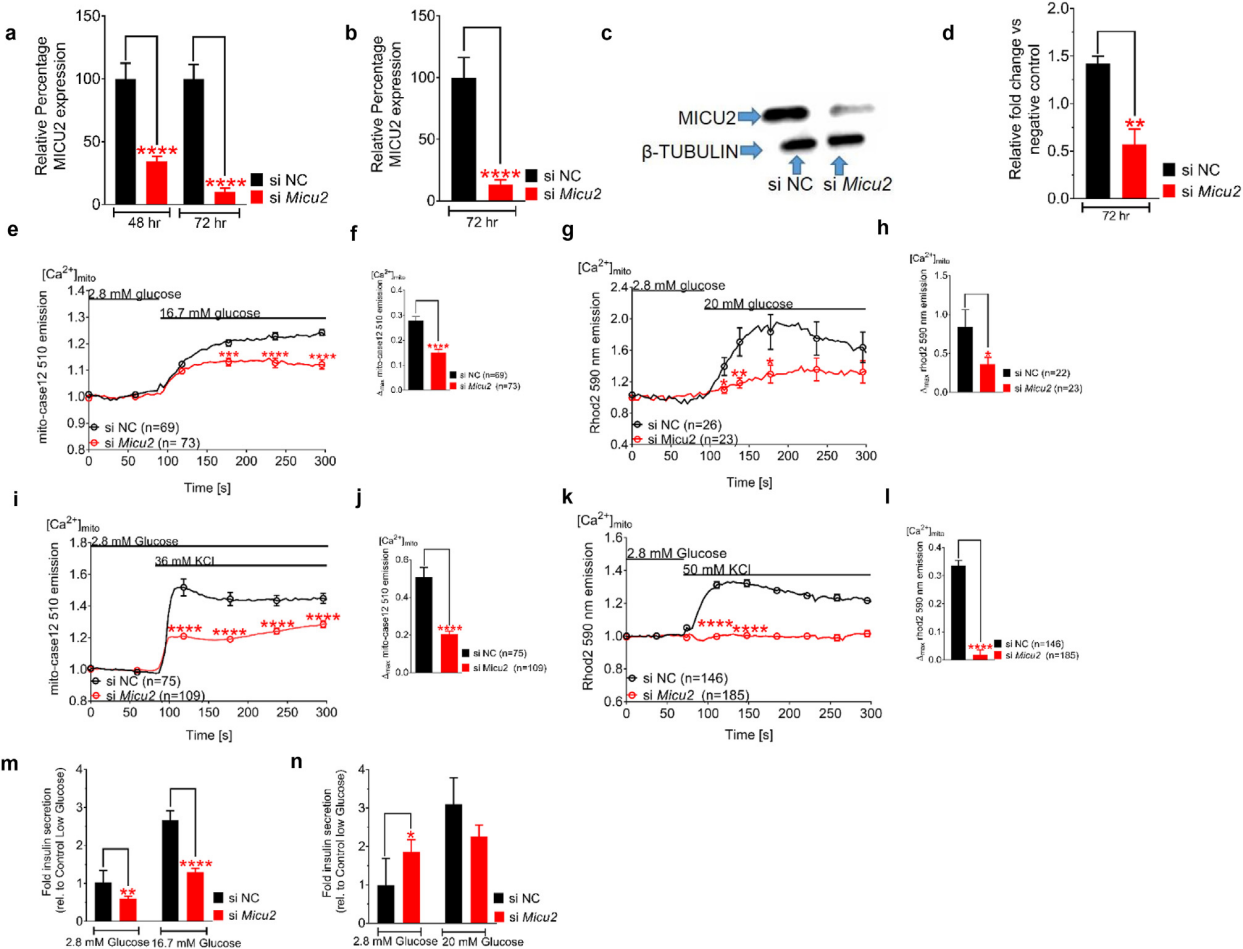
**Knockdown of *MICU2* reduced mitochondrial Ca^2+^ uptake and decreased insulin secretion.***MICU2* mRNA expression in INS-1 832/13 cells (A) and EndoC-βH1 cells (B) after treatment with scrambled or MICU2 siRNA. MICU2 protein expression (C) and quantification of Western blotting (D) in EndoC-βH1 cells. Mitochondrial Ca^2+^ ([Ca^2+^]_mito_) determined by mito-case12 and Rhod2, respectively, in MICU2-silenced INS-1 832/13 (E and F) and EndoC-βH1 cells (G and H) after glucose stimulation. E and G show average traces while F and H show differences in maximal mitochondrial Ca^2+^ levels between control and MICU2-silenced cells. [Ca^2+^]_mito_ by mito-case12 and Rhod2, respectively, in MICU2-silenced INS-1 832/13 (I and J) and EndoC-βH1 cells (K and L) after stimulation with KCl; average traces (I and K) and differences in maximal [Ca^2+^]_mito_ (J and L) between control and MICU2-silenced cells are shown. Insulin secretion in INS-1 832/13 (M) and EndoC-βH1 cells (N); secretion after 1 h of incubation was normalized to levels in cells treated with scrambled siRNA at 2.8 mM of glucose. Data are from at least three independent experiments 72 h after addition of siRNA unless otherwise stated. Mean ± SEM are given. Comparisons were made with an unpaired two-tailed Student's t-test. ∗p < 0.05, ∗∗p < 0.01, ∗∗∗p < 0.001, and ∗∗∗∗p < 0.0001. See also Fig. S1.

To understand the role of MICU2 in stimulus-secretion coupling in pancreatic β cells, we silenced *MICU2* in INS-1 832/13 cells, a rodent-derived insulinoma cell line [20], and in EndoC-βH1 cells, a human embryonic insulin-secreting cell line [21,30]. Our experiments revealed that after 48 or 72 h, small interfering (si) RNA treatment was sufficient to significantly reduce the expression of *MICU2* at the mRNA and protein level in both cell lines (Figure 1A–D). Notably, the expression of *MCU* and *MICU1* was unaffected by *MICU2* silencing (Supplementary Figs. 1A–B).

Given the proposed function of MICU2 in the uniporter holocomplex, we examined the uptake of Ca^2+^ into the mitochondria upon *MICU2* knockdown. To this end, we transfected INS-1 832/13 cells with a genetically encoded Ca^2+^ biosensor targeted to the mitochondrial matrix, mito-case12 [31]. Our experiments revealed that 16.7 mM of glucose raised [Ca^2+^]_mito_ and that this increase was attenuated by 48% after silencing of *MICU2* (*p* < 0.0001; Figure 1E–F). Similar findings were observed in EndoC-βH1 cells using another reporter, Rhod2, as these cells are difficult to transfect with plasmids [32]. In these cells, 20 mM of glucose triggered a [Ca^2+^]_mito_ increase that was attenuated by 57% upon *MICU2* silencing (*p* = 0.04; Figure 1G–H). *MICU2* knockdown reduced not only the magnitude, but also the rate of [Ca^2+^]_mito_ elevation in EndoC-βH1 cells (113 s vs 65 s to maximal [Ca^2+^]_mito_; *p* = 0.0202; Supplementary Fig. 1C). Both Rhod2 and mito-case12 showed “punctate” loading reflective of mitochondrial localization (Supplementary Figs. 1D–E). In INS-1 832/13 and EndoC-βH1 cells, evident from unnormalized mito-case12 and Rhod2 fluorescence, respectively, we observed that mitochondrial Ca^2+^ levels were also lower at basal glucose after *Micu2* silencing (Supplementary Figs. 2A–B).

Increasing extracellular K^+^ to 36 mM depolarized the plasma membrane and activated voltage-dependent Ca^2+^ channels. This resulted in a robust increase in [Ca^2+^]_mito_ in control cells, while there was only a minimal response in *MICU2*-silenced INS-1 832/13 cells (*p* < 0.0001; Figure 1I–J). Thus, the diminished mitochondrial response to elevated glucose (Figure 1E–H) could not simply be ascribed to decreased coupling between glucose metabolism and plasma membrane depolarization. EndoC-βH1 cells behaved similarly to rodent insulinoma cells, in which the maximal [Ca^2+^]_mito_ elevation following depolarization with 50 mM of KCl was diminished by 90% in *MICU2*-silenced cells (*p* < 0.0001; Figure 1K–L). This recording was performed simultaneously with the measurement of [Ca^2+^]_c_ shown in Supplementary Fig. 3B.

Our data suggest that MICU2 plays a role in [Ca^2+^]_mito_ homeostasis in insulin-secreting cell lines, which would be predicted to impact hormone secretion. Indeed, we found that GSIS was reduced by 41% and 51% at 2.8 (*p* = 0.0023) and 16.7 mM of glucose (*p* < 0.0001), respectively, in *MICU2*-deficient INS-1 832/13 cells (Figure 1M). In *MICU2*-deficient EndoC-βH1 cells, basal insulin secretion at 2.8 mM of glucose increased by 46% (*p* = 0.0245; Figure 1N). However, secretion at 20 mM of glucose did not increase; accordingly, the net fold change (2.8 mM–20 mM of glucose) in insulin secretion was markedly and significantly reduced (1.4-fold vs 3.1-fold; *p* = 0.0018).

### 3.2 MICU2 deficiency impaired bioenergetic functions in insulin-secreting cells

**Figure 2:**
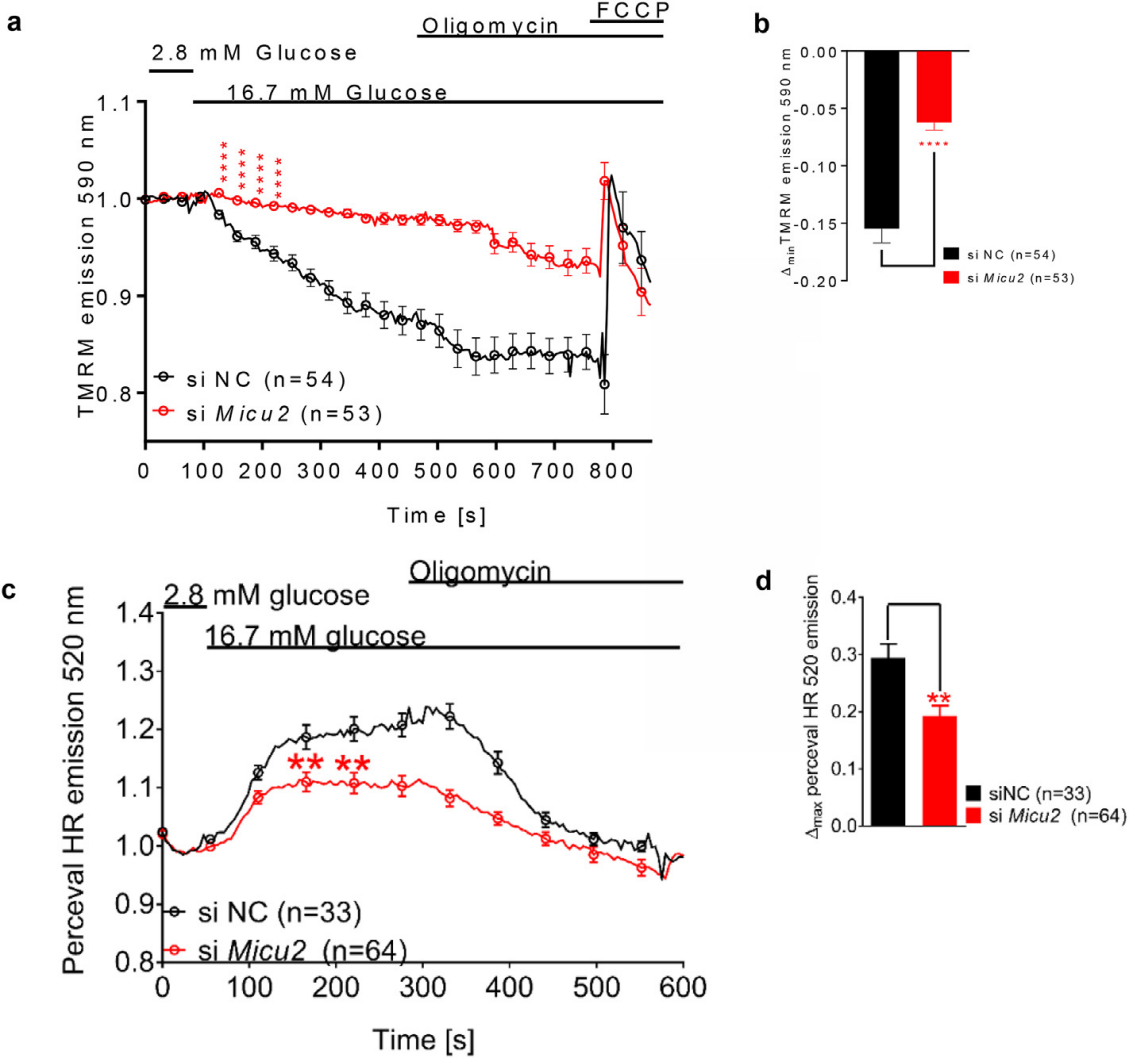
**Bioenergetic consequences of *MICU2* knockdown in INS-1 832/13 cells**. Glucose-induced mitochondrial hyperpolarization of the inner mitochondrial membrane upon *MICU2* knockdown in INS-1 832/13 cells measured by TMRM (A and B); average traces (A) and difference in maximal hyperpolarization (B) between control and *MICU2*-silenced cells are shown. The cytosolic ATP/ADP ratio in response to an elevation in glucose from 2.8 to 16.7 mM upon *MICU2* knockdown in single INS-1 832/13 cells monitored with Perceval HR (C and D); average traces (C) and difference in the maximal increase in the ATP/ADP ratio (D) between control and *MICU2*-silenced cells are shown. Data are from at least three independent experiments 72 h after adding siRNA. Mean ± SEM are given. Comparisons were made with an unpaired two-tailed Student's t-test. ∗∗*p* < 0.01 and ∗∗∗∗*p* < 0.0001.

The mitochondrial membrane potential Δψ_m_ is an essential facet of mitochondrial bioenergetics underlying both ATP synthesis and Ca^2+^ uptake into the mitochondrial matrix via MCU [33]. The mitochondrial membrane potential is influenced by uptake of Ca^2+^ into the matrix [34]. Given the potential regulatory role of MICU2 in this process and the observed attenuation of mitochondrial Ca^2+^ uptake, we monitored the relative changes in Δψ_m_ with tetramethylrhodamine methyl ester (TMRM) in quench mode. Indeed, a rise in glucose from 2.8 to 16.7 mM hyperpolarized Δψ_m_ in INS-1 832/13 cells and this response decreased by 60% after silencing of *MICU2* (*p* < 0.0001; Figure 2A–B). It therefore appears plausible that decreased TCA cycle activation after silencing was the cause of the decreased hyperpolarization. Under basal conditions, unnormalized TMRM fluorescence was stronger in *Micu2*-silenced cells, indicating that mitochondria were depolarized to a greater extent than in control cells (Supplementary Fig. 2C).

In light of this, we examined the effect of *MICU2* deficiency on glucose-stimulated changes in the cytosolic ATP/ADP ratio using live cell imaging of INS-1 832/13 cells expressing the fluorescent ATP/ADP reporter Perceval HR [25]. We found that knockdown of *MICU2* significantly reduced the increase in the cytosolic ATP/ADP ratio upon stimulation with 16.7 mM of glucose (Figure 2C), with the maximal cytosolic ATP/ADP ratio increase diminished by 36% after stimulation with 16.7 mM of glucose in *MICU2*-silenced INS-1 832/13 cells (*p* = 0.0013; Figure 2C–D). However, as previously stated, the decreased [Ca^2+^]_mito_ response to elevated glucose could not be solely ascribed to decreased bioenergetic coupling, since it was also apparent when KCl was elevated. Basal levels of ATP production also appeared to be diminished as evident from reduced unnormalized Perceval HR fluorescence at 2.8 mM of glucose (Supplementary Fig. 2D).

### 3.3 MICU2 controlled both mitochondrial and cytosolic Ca^2+^ entry

**Figure 3:**
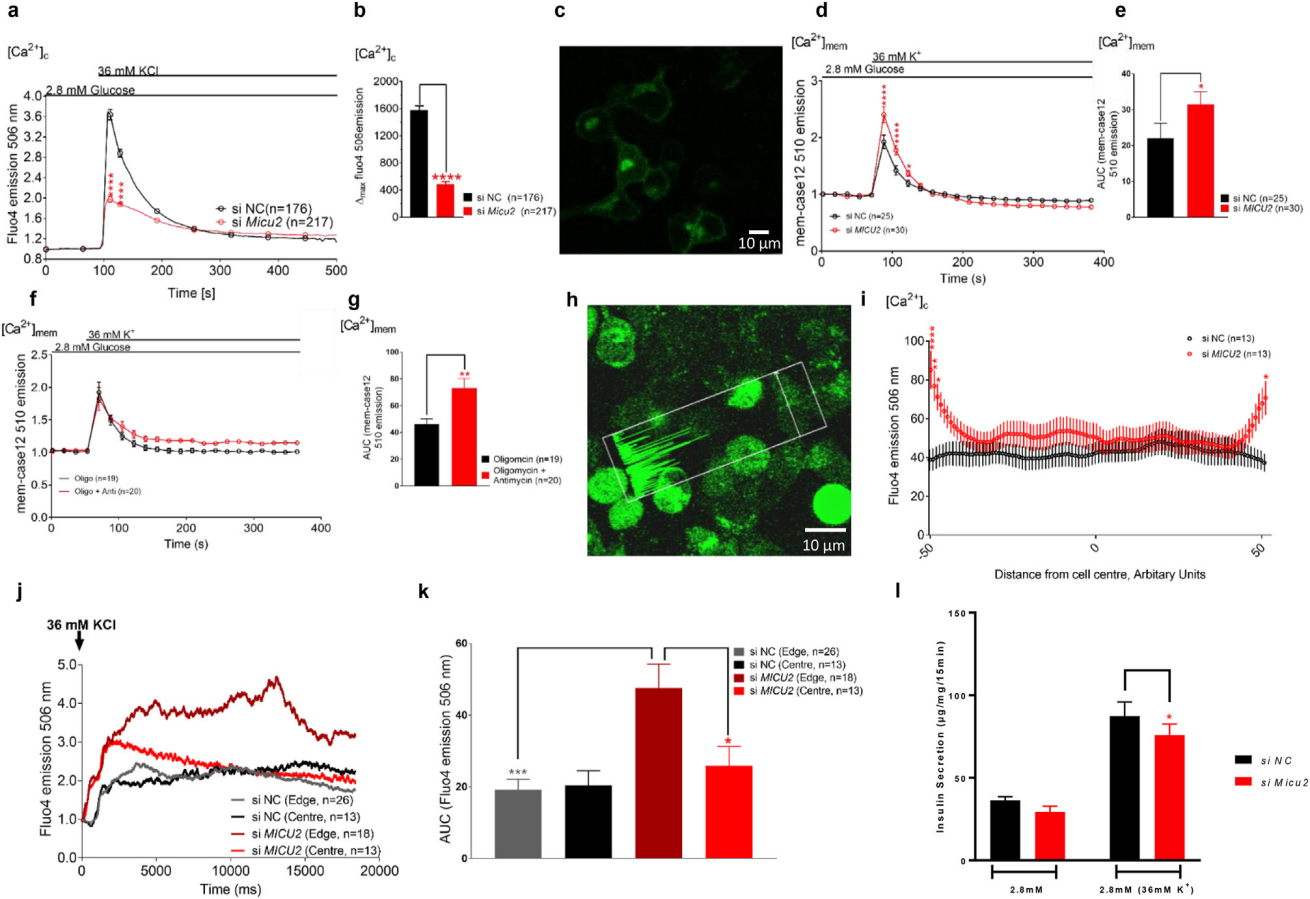
**Cytosolic and subplasmalemmal Ca^2+^ levels in response to KCl stimulation were perturbed in *MICU2*-silenced INS-1 832/13 cells**. Cytosolic Ca^2+^ levels ([Ca^2+^]c) in MICU2-silenced INS-1 832/13 cells (and scramble control) upon stimulation with 36 mM of KCl as measured by Fluo4 (A and B). Average traces are shown in A, while B shows differences in maximal Fluo4 signals. (C) Image of mem-Case12 staining, n. b. specific membrane localization. Submembrane Ca^2+^ levels of [Ca^2+^]_mem_ in *MICU2*-silenced INS-1 832/13 cells upon stimulation with 36 mM of KCl measured using mem-case12 (D and E); average traces are shown in D, while E shows iAUC quantification of KCl's effect on [Ca^2+^]_mem_. In (F and G), similar experiments are shown but using oligomycin and antimycin to block mitochondrial Ca^2+^ uptake prior to KCl addition, with oligomycin applied alone as a control. Mean ± SEM are given. (H) Example of line scan selection: the white arrow through the center of the cell is the only part of the field of view that was imaged; the accompanying trace within the white rectangle shows the fluorescence signal for each corresponding point along the arrow. (I) Mean Ca^2+^ intensity across INS-1 832/13 cells ∼500 ms after initiation of response to 36 mM of KCl. Cells had different sizes, so the data were interpolated to standardize each cell to a set number of points, allowing for comparison. The X axis represents the cell axis moving from one side of the plasma membrane (−50) through the center of the cell (0) to the opposite side of the plasma membrane (50). Points close to the extremities represent the submembrane region. (J) Average trace of cytosolic Ca^2+^ at cell edge and cell center in *MICU2*-silenced INS-1 832/13 cells (and scramble control). (K) iAUC quantification of [Ca^2+^]_c_ at the cell edge and cell center in *MICU2*-silenced INS-1 832/13 cells (and scramble control). Subplasmalemmal Ca^2+^ levels ([Ca^2+^]_mem_) in *MICU2*-silenced INS-1 832/13 cells (and scramble control) upon stimulation with 36 mM of KCl. (L) Insulin secretion in control and *Micu2*-silenced INS-1 832/13 cells after 15 min of incubation in 36 mM of KCl. Comparisons were made with an unpaired two-tailed Student's t-test. In 3L, statistical comparisons were made by a paired two-tailed Student's t-test since variability in short-term incubations was greater. ∗*p* < 0.05, ∗∗*p* < 0.01, ∗∗∗*p* < 0.001, and ∗∗∗∗*p* < 0.0001.

In the next set of experiments, we examined whether MICU2 also influences bulk [Ca^2+^]_c_ in insulin-secreting cells. Glucose-induced Ca^2+^ oscillations were reduced in *Micu2-*silenced INS-1 832/13 cells (Supplementary Fig. 3A). However, as this could be the result of lower ATP production rather than a direct effect of MICU2 on [Ca^2+^]_c_ dynamics, the cells were then depolarized non-metabolically by adding 36 mM of KCl. We predicted that depolarization-evoked [Ca^2+^]_c_ increases would be more pronounced in *MICU2*-silenced cells due to reduced buffering by the mitochondria. Surprisingly, we observed that the maximal [Ca^2+^]_c_ increase upon stimulation with 36 mM of KCl decreased by 48% in *MICU2*-silenced INS-1 832/13 cells (*p* < 0.0001; Figure 3A–B). Similar results were obtained in EndoC-βH1 cells, in which silencing of *MICU2* led to a 41% reduction in the maximal [Ca^2+^]_c_ elevation upon 50 mM of KCl stimulation (*p* = 0.0019; Supplementary Figs. 3B–C). This implied that the role of MICU2 in this paradoxical regulation of [Ca^2+^]_c_ was conserved in β cells of both rat and human origin. The measurement of [Ca^2+^]_c_ was performed simultaneously with the Rhod2 imaging shown in Figure 1K.

Given our findings, we hypothesized that the reduced depolarization-evoked [Ca^2+^]_c_ increases after *MICU2* knockdown (and mitochondrial depolarization) were due to reduced net Ca^2+^ entry across the plasma membrane (diminished entry or enhanced extrusion). To functionally elucidate this, we imaged INS-1 832/13 cells using a genetically encoded Ca^2+^ biosensor targeted to the inner leaflet of the plasma membrane mem-case12 [[35], [36], [37]]. Figure 3C shows the sensor's plasma membrane localization allowing the subplasmalemmal-free Ca^2+^ concentration ([Ca^2+^]_mem_) to be determined. If mitochondrial Ca^2+^ sequestration was indeed facilitating net Ca^2+^ entry into the cell by removing Ca^2+^ from the subplasma membrane compartment, then it would be predicted that cells with defective mitochondrial Ca^2+^ transport would show enhanced subplasmalemmal Ca^2+^ elevation in response to depolarization by KCl. Indeed, the maximal [Ca^2+^]_mem_ elevation was 20% greater in *MICU2*-deficient INS-1 832/13 cells than in control cells (*p* = 0.0118; Figure 3D). The incremental area under the curve (iAUC) was also 43% greater in the *MICU2*-deficient cells (*p* = 0.015 vs control; Figure 3E).

To establish whether this effect on [Ca^2+^]_mem_ could be accounted for by restricting MCU activity, we used an alternative strategy to inhibit mitochondrial Ca^2+^ transport [38]. In the presence of the ATP synthase inhibitor oligomycin, ATP is maintained by glycolysis and Δψ_m_ is retained, or even elevated, allowing mitochondrial Ca^2+^ transport to occur normally. Further adding an electron transport inhibitor such as antimycin rapidly collapses Δψ_m_ with no a priori effect on (glycolysis-maintained) cytosolic ATP production. Mitochondrial Ca^2+^ transport, however, being dependent upon Δψ_m_, is abolished [38]. We found that although treating control INS-1 832/13 cells with oligomycin/antimycin did not increase the maximal [Ca^2+^]_mem_ elevation, the Ca^2+^ concentration remained elevated longer at the membrane, with a delayed return to the basal state (Figure 3F). This was reflected by the iAUC of the effect increasing by 58% compared to oligomycin control (*p* = 0.0026; Figure 3G). Thus, this treatment had a comparable effect on [Ca^2+^]_mem_ as *MICU2* deficiency. An important proviso is that the capacity of INS-1 832/13 cells to accelerate glycolysis in the presence of oligomycin is limited by their low expression of lactate dehydrogenase [39]; therefore, experiments were performed during the time line before which ATP depletion became significant as indicated by the unperturbed mitochondrial membrane potential during this time (data not shown). Furthermore, using oligomycin alone as a control implied that any effect upon ATP production could not explain the reduced extrusion of [Ca^2+^]_mem_ in oligomycin/antimycin-treated INS-1 832/13 cells and must therefore have been due to a decrease in mitochondrial Ca^2+^ uptake.

To further examine whether subplasmalemmal Ca^2+^ elevations occur when mitochondrial Ca^2+^ uptake is abrogated, we imaged INS-1 832/13 cells loaded with Fluo4 using the line scan configuration. This allows repeated imaging across a line through a cell axis as opposed to the entire field of view (Figure 3H). Crucially, this method allows the higher temporal resolution required for tracking Ca^2+^ signal propagation following membrane depolarization. To this end, cell(s) were selected, after which the starvation medium was rapidly replaced with medium containing 36 mM of KCl and the recording started simultaneously. It was expected that MICU2-deficient cells would show enhanced subplasmalemmal Ca^2+^ elevation in response to depolarization by KCl, reflected by a stronger fluorescence signal intensity at the edge of the line scan. We found that, upon KCl-induced depolarization, Ca^2+^ accumulated at the subplasma membrane compartment in si*MICU2*-treated INS-1 832/13 cells (Figure 3I–K) with a 1.85-fold higher Ca^2+^ signal (measured as iAUC) at the edge of the cell compared to its center (*p* = 0.0266). Furthermore, there was a 2.5-fold higher Ca^2+^ response at the submembrane compartment in *MICU2*-deficient INS-1 832/13 cells compared to controls (*p* = 0.0006 vs controls; Figure 3K). In contrast, there was no significant difference in the Ca^2+^ signal between the edge and center in the control cells (*p* = 0.8027; Figure 3K). Note that when analyzing the line scan data, the nucleus was avoided when selecting the “center” of the cell.

To ensure that the effects on [Ca^2+^]_mem_ were not the result of off-target effects of *Micu2* siRNA on voltage-gated Ca^2+^ channel expression, expression of L-type and P/Q-type Ca^2+^ channels in *Micu2-*silenced INS-1 832/13 cells were determined by qPCR: no difference in expression was observed (Supplementary Fig. 4A). We repeated the line scan experiments in EndoC-βH1 cells but were unable to see any difference between control and *Micu2*-silenced cells (Supplementary Fig. 4B). Using mem-case12 in Endo C-βH1 cells was not possible due to the cytotoxic effects of the transfection protocols.

Collectively, these observations support a model in which *MICU2*-deficient mitochondria in the vicinity of the plasma membrane are less capable of removing Ca^2+^ from this compartment. Our results thus indicate not only that MICU2 plays a critical role in controlling MCU and Ca^2+^ homeostasis, but also that mitochondria play a critical role in buffering local subplasmalemmal Ca^2+^ as previously observed [40], facilitating net Ca^2+^ entry into the cell. This may occur either by decreasing cytosolic Ca^2+^-mediated desensitization of voltage-dependent Ca^2+^ channels in the plasma membrane [41,42] or alternatively by restricting Ca^2+^ efflux by plasma membrane Ca^2+^-ATPases [43,44]. The latter was unlikely in the present experimental situation since *MICU2* deficiency inhibited an increase in the ATP/ADP ratio, which will limit access to ATP for the ATPases. To assess how this change in Ca^2+^ dynamics ultimately affects insulin secretion, INS-1 832/13 cells were exposed to 36 mM of KCl for 15 min: insulin secretion was reduced in *MICU2*-silenced cells (Figure 3L).

### 3.4 *Micu2*-deficient mice showed defective stimulus-secretion coupling and insulin secretion

**Figure 4:**
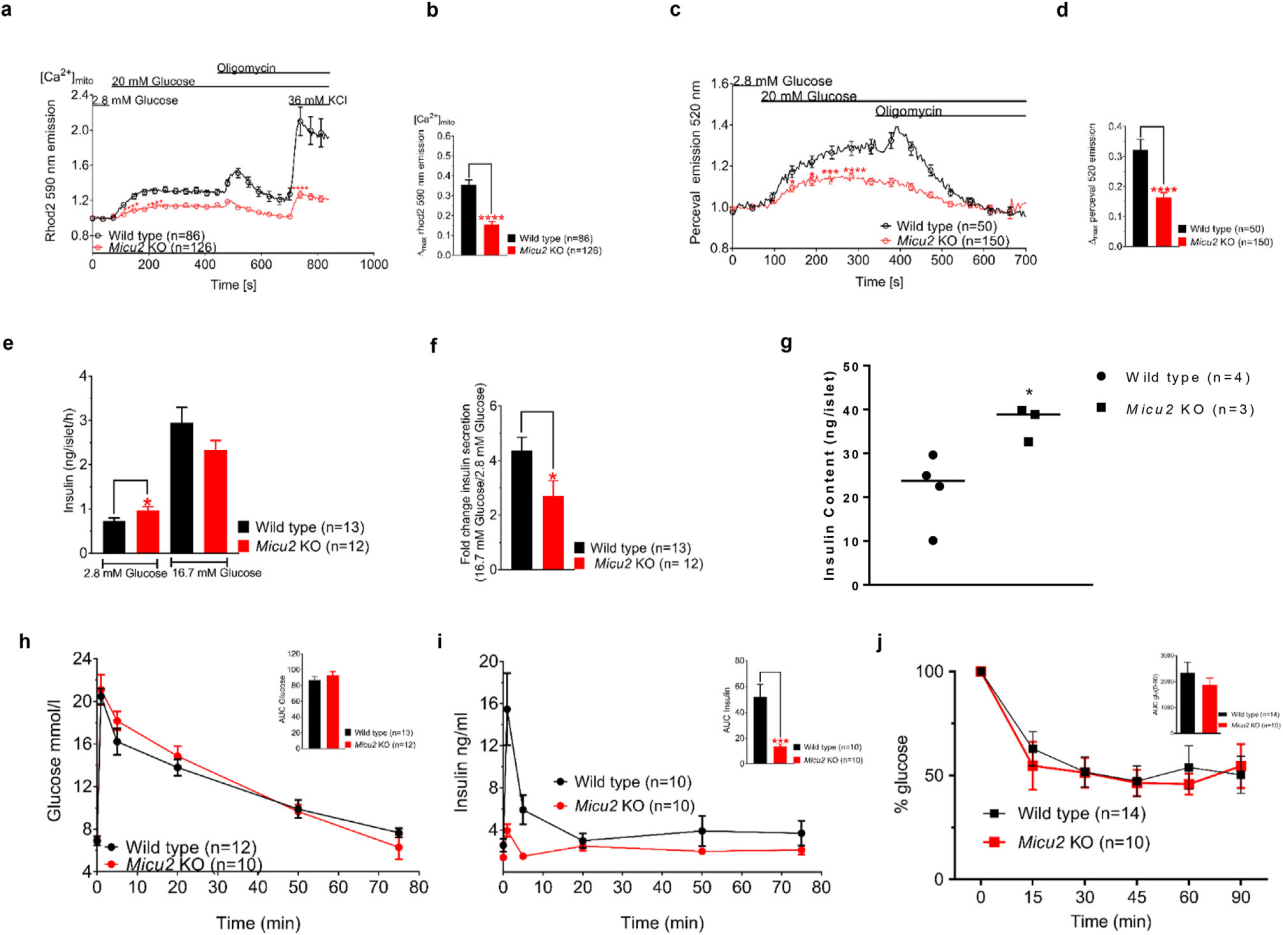
***Micu2* knockout mice exhibited deficiencies in mitochondrial Ca^2+^ homeostasis, ATP/ADP ratio, and insulin secretion**. Mitochondrial Ca^2+^ levels [Ca^2+^]_mito_ in islets isolated from *Micu2* knockout (KO) and wild-type mice measured with Rhod2 after stimulation with 20 mM of glucose (A and B). Average traces are shown in (A) while (B) shows differences in maximal Rhod2 signals. A total of 40 islets from 7 wild-type mice and 42 islets from 6 *Micu2* KO mice were assayed in response to 20 mM of glucose and 36 mM of KCl. Changes in the cytosolic ATP/ADP ratio in response to 20 mM of glucose in *Micu2* KO and wild-type islets were determined by Perceval (C) n = 14 and 27 islets from 4 to 6 wild-type and KO islets, respectively. Average traces are shown in (C) while (D) shows differences in maximal Perceval signals. Insulin secretion from *Micu2* KO and wild-type islets at 2.8 and 16.7 mM of glucose after 1 h of incubation (E); (F) shows the fold response of insulin. (G) Insulin content in *Micu2* KO and wild-type islets. Plasma glucose (H) and insulin (I) levels following an intravenous glucose tolerance test in *Micu2* KO and wild-type mice; n denotes the number of mice. Insets show the area under the curve for glucose (AUC; H) and insulin (J; T = 0–5 min). Data are from at least three independent experiments. (I) Insulin tolerance test (ITT) in *Micu2* KO and wild-type mice. Glucose values are expressed as the percentage of glucose in which glucose at 0 min was 100%; n denotes the number of mice. Inset shows the AUC. Mean ± SEM are given. Comparisons of imaging and insulin secretion were made with an unpaired two-tailed Student's t-test (∗*p* < 0.05 and ∗∗∗∗*p* < 0.0001) and the Mann–Whitney U-test (∗∗∗*p* < 0.001) for data from IVGTT.

Our experiments suggested that perturbation of MICU2 led to reduced mitochondrial Ca^2^ uptake, resulting in perturbed Ca^2+^ entry into the cell and, consequently, defective insulin secretion in both rodent-derived INS-1 832/13 and human EndoC-βH1 cell lines. To understand the consequences of MICU2 deficiency in a more physiological setting, we used a global *Micu2* knockout mouse [23]. As previously observed in insulin-secreting cell lines, glucose-stimulated (20 mM) maximal [Ca^2+^]_mito_ elevation was diminished by 57% in *Micu2* knockout islets compared to wild-type islets (*p* < 0.0001; Figure 4A–B). Note that the KCl response was also diminished (Figure 4A), similar to that observed in the cell lines. These results suggested that MICU2 most likely plays a similar role in maintenance of [Ca^2+^]_mito_ homeostasis in primary cells to that observed in cell lines.

Furthermore, as [Ca^2+^]_mito_ homeostasis is critical for cellular bioenergetics, we speculated that the *Micu2* knockout might perturb the cytosolic ATP/ADP ratio in primary cells, as was seen in the insulin-secreting cell lines. Therefore, in the next set of experiments, we used an adenovirus expressing the biosensor Perceval, originally described as an ATP/ADP sensor [26], but later shown to report cytoplasmic ATP in mouse islet cells [45]. The maximal Perceval signal was diminished by 50% in *Micu2* knockout islets compared with wild-type islets upon stimulation with 20 mM glucose (*p* < 0.0001; Figure 4C–D). A likely consequence of an abrogated increase in the ATP/ADP ratio in pancreatic β cells is impaired insulin secretion. Batch incubations of *Micu2* knockout islets demonstrated that basal (2.8 mM of glucose) insulin secretion was significantly higher compared to that in their wild-type counterparts (*p* = 0.0427; Figure 4E). However, *Micu2* knockout islets failed to release insulin in response to high glucose as efficiently as wild-type islets as illustrated by a 50% reduction in the fold response of insulin secretion (16.7–2.8 mM of glucose; *p* = 0.0362; Figure 4F). Notably, insulin content was found to be increased in *Micu2* knockout islets compared with wild-type islets (*p* < 0.05; Figure 4G). This effect was most likely due to the accumulation of insulin that was not released since *INS1* mRNA expression was similar in wild-type and *Micu2* knockout islets (Supplementary Fig. 5A). Supplementary Fig. 5B shows the islet secretion data normalized to the insulin content derived from the analysis shown in Figure 4G: in vitro results shown this way reflect the in vivo observations, in that stimulated insulin secretion was abrogated while basal secretion was unchanged.

To ensure that any effects were not due to changes in the expression of the mitochondrial complexes, we quantified the protein expression of complexes I–V by Western blotting. There were no significant differences between wild-type and *Micu2* knockout islets (Supplementary Figs. 5C–D). Our experiments thus suggested that stimulus-secretion coupling in β cells in *Micu2* knockout mice was defective and underlied perturbed GSIS, reminiscent of that found in EndoC-βH1 cells (Figure 1N).

In the next set of experiments, we examined the consequences of *Micu2* deficiency on whole-body glucose homeostasis. Glucose elimination was not significantly different between the *Micu2* knockout and wild-type mice (Figure 4H). However, an intravenous glucose tolerance test (IVGTT) revealed a marked reduction in the initial release of insulin (*p* = 0.0003; Figure 4I): we observed a 74% and 70% reduction in the plasma insulin levels at 1 and 5 min after glucose administration, respectively, in *Micu2* knockout mice compared to wild-type mice. Because insulin secretion was impaired while glucose elimination was unaltered in *Micu2* deficiency, we performed an insulin tolerance test (ITT). Both strains of mice responded robustly to insulin, with decreased plasma glucose levels (Figure 4J). These experiments thus demonstrated that the initial release of insulin in *Micu2* knockout mice was reduced, likely caused by a stimulus-secretion coupling defect in pancreatic β cells. Hence, *Micu2* knockout mice exhibited deficient GSIS both in vitro and in vivo.

### 3.5 MICU2 regulation of Ca^2+^ in mitochondria, cytosol, and subplasmalemmal space was conserved between cell types

Previous studies in neuronal cells showed that inhibition of ATP generation and dissipation of mitochondrial membrane potential led to reduced [Ca^2+^]_c_ upon stimulation with glutamate or 50 mM of KCl [46]. Therefore, having shown the role of MICU2 in regulating [Ca^2+^]_mito_ and [Ca^2+^]_c_ in insulin-secreting cells, we asked whether this mechanism may also be observed in other cell types. High-throughput gene expression assays previously demonstrated that the human embryonic kidney-derived HEK-293T cell line robustly expresses levels of *MICU2* mRNA [47]. It has been shown that this cell line also expresses voltage-dependent Ca^2+^ channels and exhibits endogenous Ca^2+^ currents [48,49]. We therefore used this cell line to address the role of *MICU2* in cellular Ca^2+^ homeostasis.

**Figure 5:**
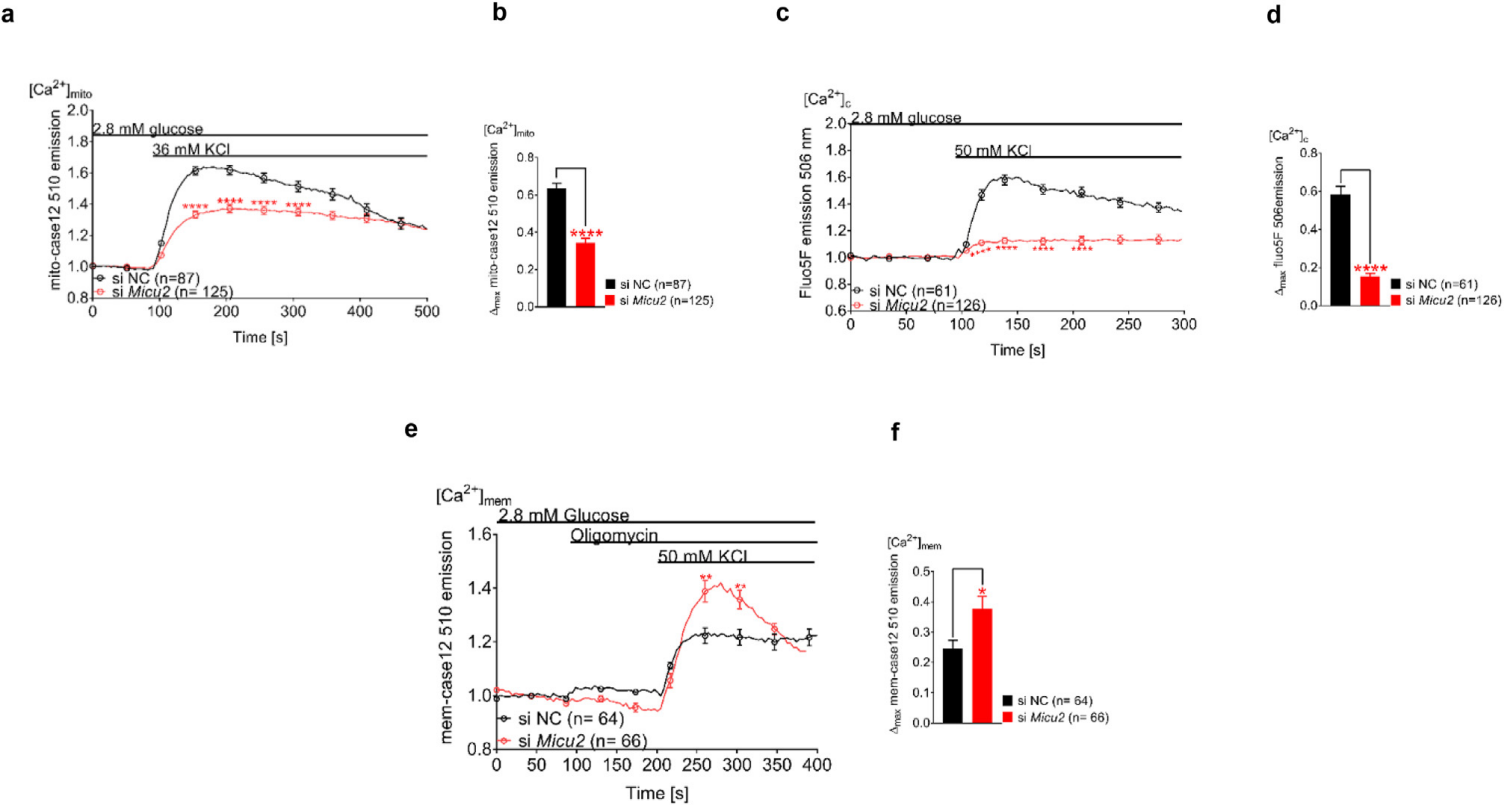
**MICU2 played a similar role in Ca^2+^ homeostasis in HEK-293T cells as observed in insulin-secreting cells**. Mitochondrial Ca^2+^ ([Ca^2+^]_mito_) determined by mito-case12 in *MICU2*-silenced HEK-293T cells stimulated with 36 mM of KCl (A and B); (A) shows average traces while (B) shows differences in the maximal mitochondrial Ca^2+^ response. Cytosolic Ca^2+^ levels ([Ca^2+^]_c_) determined by Fluo5F in *MICU2*-silenced HEK-293T cells upon stimulation with 50 mM of KCl (C and D); average traces are shown in (C) while (D) shows differences in maximal Fluo5F signals. Subplasmalemmal Ca^2+^ levels ([Ca^2+^]_mem_) in *MICU2*-silenced HEK-293T cells upon stimulation with 50 mM of KCl (E and F); average traces are shown in (E) while (F) shows differences in maximal mem-case12 signals. Data are from at least three independent experiments 72 h after adding siRNA. Mean ± SEM are given. Comparisons were made with an unpaired two-tailed Student's t-test. ∗*p* < 0.05, ∗∗*p* < 0.01, and ∗∗∗∗*p* < 0.0001. See also Fig. S3.

*MICU2* was effectively silenced on the mRNA and protein levels in HEK-293T cells (Supplementary Figs. 6A–C). Measurements of [Ca^2+^]_mito_ with mito-case12 demonstrated that stimulation with 36 mM of KCl increased [Ca^2+^]_mito_ in HEK293T cells and that this response was attenuated by 46% after silencing of *MICU2* (*p* < 0.0001; Figure 5A–B). In addition, time to maximal [Ca^2+^]_mito_ peak in *MICU2*-silenced cells was 126 s compared to 107 s in controls after 36 mM of KCl stimulation (*p* = 0.0087; Supplementary Fig. 6D). [Ca^2+^]_c_ measurements using Fluo5F showed that membrane depolarization by 50 mM KCl triggered a [Ca^2+^]_c_ increase and the maximal [Ca^2+^]_c_ elevation was reduced by 74% in *MICU2*-silenced HEK-293T cells compared to control cells (*p* < 0.0001; Figure 5C–D). For experiments measuring [Ca^2+^]_mem_ using mem-case12, oligomycin was added to ensure that any effects of *MICU2* silencing were independent from the resultant bioenergetic effects of increased [Ca^2+^]_mito_. The same was not done in the experiments with INS-1 832/13 cells due to ATP production in the β cell being finely tuned to the extracellular glucose concentration as a consequence of the low-affinity enzymes GLUT2 and glucokinase and downstream dissipative pathways (high leak current) [50]. This means that at low glucose, metabolic flux and ATP production in INS-1 832/13 cells are low compared to HEK293T cells. Therefore, any effect of increased [Ca^2+^]_mito_ on ATP production would be unlikely to reach the threshold to affect K_ATP_ channel activity, meaning using oligomycin was not necessary. Reminiscent of the findings in INS-1 832/13 cells, [Ca^2+^]_mem_ elevation upon 50 mM of KCl stimulation increased by 35% in *MICU2*-silenced HEK293T cells compared to the control cells (*p* = 0.0103; Figure 5E–F). Thus, these data demonstrate a conservation of the regulation of [Ca^2+^]_mito_ and [Ca^2+^]_c_ by MICU2 in different cell types.

## 4 Discussion

It is undisputed that an increase in [Ca^2+^]_c_ triggers insulin secretion as well as secretion of most other hormones, but it has also been recognized that such a rise in itself does not sustain insulin secretion [51,52]. Cytosolic Ca^2+^ is further transported into the mitochondrial matrix, and an elevation in free matrix Ca^2+^ has been considered to trigger additional regulatory factors [53]. Prior to the molecular identification of the components of the uniporter holocomplex [19,54,55], indirect approaches were required to investigate the effect of modulating [Ca^2+^]_mito_, none of which were entirely satisfactory. Removal of external Ca^2+^ [56] affects multiple non-mitochondrial processes, not least exocytosis itself. Moreover, attempts to dampen changes in [Ca^2+^]_mito_ by increasing the matrix Ca^2+^-buffering capacity by targeting exogenous Ca^2+^-binding proteins to the matrix [5] or loading cells with Ca^2+^-chelator 1,2-bis(o-aminophenoxy)ethane-*N,N,N′,N′-*tetraacetic acid (BAPTA) [57] each succeeded in reducing the bioenergetic response to elevated glucose, but concerns remain about toxicity associated with this treatment [50].

The molecular characterization of the uniporter [19,54,55,58] as well as a number of accessory proteins such as MICU1, 2, 3, and EMRE [19,55,59] opened the possibility of genetic manipulation of the uniporter's components [60]. It was shown that silencing of *Mcu* abrogates the increase in [Ca^2+^]_mito_ and the cytosolic ATP/ADP ratio in response to glucose in mouse β cells [61]. Another study demonstrated that silencing of *MCU* in clonal INS-1-E cells resulted in reduced nutrient-induced hyperpolarization of the inner mitochondrial membrane, ATP production, and GSIS [9]. Moreover, knockdown of *MICU1* leads to reductions in [Ca^2+^]_mito_, cytosolic ATP levels, and GSIS in clonal insulin-secreting cells [8]. However, the role of MICU2 remains more enigmatic. MICU2 is a peripheral membrane protein associated with the mitochondrial inner membrane and facing the intermembrane space. The absence of MICU2 has been associated with reduced Ca^2+^ uptake in vivo in mouse liver, though silencing in this system also leads to a decrease in the abundance of the pore-forming subunit MCU and the complex [19]. Observations in other cell systems suggested that the accessory protein may have a regulatory role in Ca^2+^ uptake into mitochondria [13,16]. MICU1 and MICU2 are thought to heterodimerize via disulfide bridges and thus regulate Ca^2+^ entry into the mitochondrial matrix via the MCU [62]. Indeed, both MICU1 and MICU2 have been shown to play an important role in setting the threshold for mitochondrial Ca^2+^ uptake in HeLa cells at variable Ca^2+^ concentrations [16]. Furthermore, loss of MICU1 or MICU2 in permeabilized HEK-293T cells has also been shown to disrupt this critical threshold for mitochondrial Ca^2+^ uptake [13,14,63,64].

Herein, using *MICU2*-deficient rodent and human insulin-producing cell lines as well as *Micu2* knockout mice, we were able to show that reduced Ca^2+^ uptake into mitochondria resulted in inhibition of GSIS. This confirmed observations in previous studies [8,9,61] showing that [Ca^2+^]_mito_ affects mitochondrial metabolism and insulin secretion. However, our experiments could not resolve how *Micu2* knockout mice maintained euglycemia despite reduced early insulin secretion. Insulin tolerance, albeit a rough estimate of insulin action in vivo, was unchanged. In fact, we previously reported that ITT is not sensitive enough to reflect changes in insulin sensitivity in the face of reduced early insulin secretion in vivo [65]. This phenotype is also somewhat similar to what was observed in β cell-specific *Mcu* knockout mice that exhibit impaired insulin secretion 5 min after intraperitoneal glucose injection with improved glucose tolerance despite normal insulin sensitivity [15]. A hyperinsulinemic euglycemic clamp, which is beyond the scope of the present study, would be required to accurately determine insulin sensitivity. Importantly, *MCU* and *MICU1* expression were unaffected by *MICU2* silencing in INS-1 832/13 cells, indicating that MICU2 plays a fundamental non-redundant role in regulating [Ca^2+^]_mito_ in β cells. However, the mechanisms by which MICU2 and [Ca^2+^]_mito_ might exert control over GSIS were unexpected and add a new dimension to how mitochondrial Ca^2+^ transport might regulate β cell function and insulin secretion.

The key observation novel in the context of β cells is that mitochondrial Ca^2+^ uptake exerts a profound stimulatory effect on the net flux of cations across the plasma membrane, either by enhancing entry or depressing efflux. Insulin-secreting cells have a high concentration of subplasma membrane mitochondria, with roughly 20% of the membrane inner surface associated with mitochondria exerting a strong Ca^2+^-buffering effect [40]. Mitochondria are dynamic organelles and submembrane Ca^2+^ buffering is reduced upon plasma membrane depolarization because of Ca^2+^-dependent remodeling of the cortical F-actin network causing translocation of subplasmalemmal mitochondria to the cell interior [40]. In line with these findings, we demonstrated that restricted mitochondrial Ca^2+^ uptake resulted in more pronounced depolarization-induced increases in Ca^2+^ in the submembrane compartment. However, under the same conditions, the global cytoplasmic Ca^2+^ increases were attenuated.

To rationalize a decreased Ca^2+^ response to plasma membrane depolarization in both the matrix and cytosolic compartments, the simplest assumption is that net Ca^2+^ influx across the plasma membrane is inhibited. Increased extrusion of Ca^2+^ is less likely given the observed decrease in the ATP/ADP ratio as a consequence of MICU2 deficiency and that extrusion is an ATP-dependent process. While the endoplasmic reticulum does take up Ca^2+^ in response to the elevated ATP/ADP ratio at high glucose, the response is too slow to account for a Ca^2+^ deficit [66]. In any case, the effect of restricted mitochondrial Ca^2+^ uptake on bulk [Ca^2+^]_c_ is seen with elevated K^+^ as well as with high glucose and is thus not directly dependent on bioenergetics. Had this effect only been observed in response to glucose, then the previously postulated positive effect of mitochondrial Ca^2+^ uptake on mitochondrial dehydrogenases would have been the most plausible explanation [67].

A paradoxical decrease in [Ca^2+^]_c_ elevation following inhibited transport of mitochondrial Ca^2+^ was previously observed in cultured neurons [38,46]. Since these studies predated the molecular identification of the MCU, mitochondrial Ca^2+^ transport was inhibited by mitochondrial depolarization in the presence of the ATP synthase inhibitor oligomycin together with a rotenone or antimycin electron transport inhibitor. The control was oligomycin alone: both control and mitochondrial depolarized cells maintained similar ATP/ADP ratios supported by glycolysis, but the [Ca^2+^]_c_ transients induced by Ca^2+^ entry through NMDA-selective glutamate receptors or voltage-dependent Ca^2+^ channels drastically reduced. In the present study, treatment with oligomycin + antimycin further confirmed a role of mitochondrial Ca^2+^ uptake in clearing subplasmalemmal Ca^2+^ in insulin-secreting cells, and the observations were similar to those in neurons [38,46]. Importantly, the net neuronal accumulation of ^45^Ca^2+^ is strongly inhibited by mitochondrial depolarization [38,46], confirming the organelle's ability to control net flux across the plasma membrane. Notably, mitochondrial Ca^2+^ uptake has also been implicated in store-operated Ca^2+^ entry, with MCU knockdown in RBL mast cells shown to reduce Ca^2+^ influx through Ca^2+^ release-activated channels [68].

It was hypothesized that mitochondria are able to deplete a subplasma membrane layer of elevated Ca^2+^ [38,46]. Voltage-dependent Ca^2+^ channels become desensitized when local Ca^2+^ accumulations are not promptly removed [44,69,70]. In addition, it has been shown in chromaffin cells, neurons, and cardiac muscle that mitochondrial sequestration of subplasmalemmal Ca^2+^ prevents desensitization of Ca^2+^ channels and thus might uphold hormone secretion [[71], [72], [73]]. Furthermore, mitochondria play an essential role in the maintenance of [Ca^2+^]_c_ amplitude wave propagation via feedback regulation of plasma membrane channel activity in diverse cellular systems, including rat cortical astrocytes and *Xenopus laevis* oocytes [74,75]. The response of the subplasmalemmal-targeted Ca^2+^ probe mem-case12 and line scan imaging in the present study provided support for the existence of such a layer of elevated Ca^2+^ [38,46] during β cell depolarization and its depletion by mitochondrial Ca^2+^ uptake. Notably, in the mem-case12 experiments, there was a difference between the effects on [Ca^2+^]_mem_ between *Micu2* silencing and treatment with oligomycin/antimycin, whereby the former caused an increase in the duration of the subplasmalemmal Ca^2+^ increase while the latter resulted in an increased magnitude of the response. This may be explained by the different mechanisms by which mitochondrial Ca^2+^ uptake is inhibited. When MICU2 is deficient, uptake at low Ca^2+^ is likely to increase as the protein plays an important role in suppressing MCU activity under these conditions [11,13,16,17]. Subsequently, when [Ca^2+^]_c_ is elevated, the mitochondrial buffering capacity decreases. In contrast, oligomycin and antimycin prevent proton transport from the mitochondrial matrix to the mitochondrial intermembrane space, increasing the matrix pH, subsequently collapsing Δψ_m_ and inhibiting Ca^2+^ entry. These two methods affect the Ca^2+^ electrochemical gradient differently and as a result the Ca^2+^ uptake kinetics, potentially explaining the differential effects on [Ca^2+^]_mem_.

Regarding line scan imaging, one could argue that due to its relatively low K_d_, Fluo4 is unsuitable for measuring [Ca^2+^]_mem_, which can reach concentrations of 10–100 μM [76]. This means there is a risk of Fluo4 saturation at the submembrane compartment. This did not seem to occur in the control, where there was little difference in the Fluo4 signal between the edge and center of the cell. In *MICU2*-silenced cells, however, there was a clear difference between the edge and center of the cell. If saturation was indeed occurring, this would imply that the observed increase in [Ca^2+^]_mem_ was in fact an underestimation. Moreover, the saturation problems were mitigated against the complementary findings using the lower-affinity mem-case12 probe (K_d_ = 1 μM) [36].

The lack of a [Ca^2+^]_mem_ effect on *MICU2*-silenced Endo C-βH1 cells may lie in differences in the composition of voltage-gated Ca^2+^ channels between the two cell lines. Notably, the L-type Ca^2+^ channel current, which is largely responsible for Ca^2+^ entry upon high K^+^ stimulation due to the rapid inactivation of other Ca^2+^ channels [77], only accounts for 20–30% of the Ca^2+^ current in Endo C-βH1 cells [78] compared to at least 60% of the Ca^2+^ current in INS-1 832/13 cells [79]. This means the [Ca^2+^]_mem_ levels attained in EndoC-βH1 cells in the line scan experiment may still be able to be cleared by *MICU2*-deficient mitochondria. Thus, any buildup of Ca^2+^ in the subplasmalemmal compartment may occur at a slower rate, evading detection in our experiments. This would also explain the smaller effect of *MICU2* silencing on depolarization-evoked [Ca^2+^]_c_ increases in EndoC-βH1.

This study shows that mitochondrial Ca^2+^ transport has additional consequences for β cells beyond facilitation of the TCA cycle, which may have varying effects on insulin secretion. On the one hand, attenuation of subplasmalemmal Ca^2+^ elevations could reduce the triggering of exocytosis in pre-docked vesicles responsible for first-phase insulin release. On the other hand, there is evidence that increased Ca^2+^ in the cytosol is required to transport reserve vesicles to the release site, at least in neuronal systems [80]. Based on the KCl-induced insulin secretion experiments, the attenuation of mitochondrial Ca^2+^ transport leads to a reduction in insulin secretion. First-phase insulin release occurs in the first 5–10 min [81]. Therefore, while there may be an increase in first-phase insulin secretion, an attenuation of the second phase likely caused by decreased transport of reserve vesicles means that insulin secretion decreases over the 15 min time course.

[Ca^2+^]_c_ increases may also occur as a consequence of endoplasmic reticulum Ca^2+^ release. Indeed, there is evidence in β cells of a basal endoplasmic reticulum Ca^2+^ leak channeled toward the mitochondria, which stimulates mitochondrial bioenergetics, priming the mitochondria for an increase in glucose [82,83]. This process has been implicated in the generation of cytosolic Ca^2+^ oscillations and GSIS [83]. Although ablating *Micu2* would also abrogate this process, it appears to be distinct from the mechanism observed herein. The reduction in [Ca^2+^]_mito_ and the associated increase in [Ca^2+^]_mem_ were more or less instantaneous upon exposure to KCl and plasma membrane depolarization as opposed to the endoplasmic reticulum Ca^2+^ leak, which seems to occur at a constant rate [82,83].

It was previously demonstrated that dynamic changes in the subplasmalemmal mitochondrial population participate in the regulation of local Ca^2+^ levels and consequently exocytosis in MIN6 cells [40]. A limitation of our study is that, at this point, we have not obtained sufficient data on whether MICU2 deficiency impacts the localization, morphology, or number of mitochondria in the submembrane compartment. Changes in any of these parameters in MICU2-deficient cells would likely also affect subplasmalemmal and cytosolic Ca^2+^ dynamics.

Our results may help resolve some of the contradictions in the concept of amplification pathways and K_ATP_-independent mechanisms of insulin secretion. The search for coupling factors responsible for K_ATP_-independent insulin secretion began after the discovery that insulin secretion could be provoked by increasing glucose even when the K_ATP_ channel was kept open by diazoxide and the plasma membrane was depolarized by high concentrations of extracellular KCl, thereby opening voltage-dependent Ca^2+^ channels [51]. Metabolic coupling factors were postulated to account for the insulinotropic effects of glucose under K_ATP_-independent conditions and explain why insulin secretion could not be sustained by an elevation of [Ca^2+^]_c_ alone [52]. However, at least the latter circumstance can be accounted for by the consequences of mitochondrial Ca^2+^ uptake that we observed herein: if Ca^2+^ is not removed from the subplasmalemmal space by mitochondria, voltage-dependent Ca^2+^ channels will be desensitized. A number of metabolites and metabolic pathways have been convincingly implicated in amplification of GSIS [84]. However, most share a similar feature: it has not been resolved how these factors stimulate exocytosis. Moreover, a number of concerns have also been raised about the bioenergetics of the formation of metabolic coupling factors [50].

## 5 Conclusions

In summary, we uncovered a crucial role of mitochondria in maintaining β cell excitability involving sequestering of Ca^2+^ into mitochondria located at the vicinity of the voltage-dependent Ca^2+^ channels in the plasma membrane that coupled with the well-established bioenergetic effects accounts for the regulatory effect of mitochondrial Ca^2+^ uptake on insulin secretion. The recent molecular characterization of the mitochondrial Ca^2+^ uniporter holocomplex holds promise for further elucidating the complexities of β cell stimulus-secretion coupling in which the mitochondria are at center stage.

## Author contributions

N.V., A.H., D.G.N., and H.M. designed the study. N.V., A.H., A.B., A.M.B., H.B., Y.S., K.K., P.S., E.C., and M.F. performed the experiments. N.V., A.H., A.M.B., and M.F. analyzed the data. V.M. provided the *Micu2* knockout mice. A.T., V.M., D.G.N., and H.M. provided critical insights to the project. N.V., A.H., A.T., V.M., D.G.N., and H.M. wrote the manuscript.

## Study approval

All of the experimental animal protocols were approved by the local animal ethical committee of Lund/Malmö and complied with the guidelines of the Federation of European Laboratory Animal Science Association (FELASA).
