# Supplementary material for: Mitochondrial clearance of calcium facilitated by MICU2 controls insulin secretion
Source: Mol Metab. 2021 Apr 28;51:101239. doi: 10.1016/j.molmet.2021.101239 (PMC8163986; doi:10.1016/j.molmet.2021.101239)
Supplement: Multimedia component 1 [file mmc1.pptx]

## Slide 1
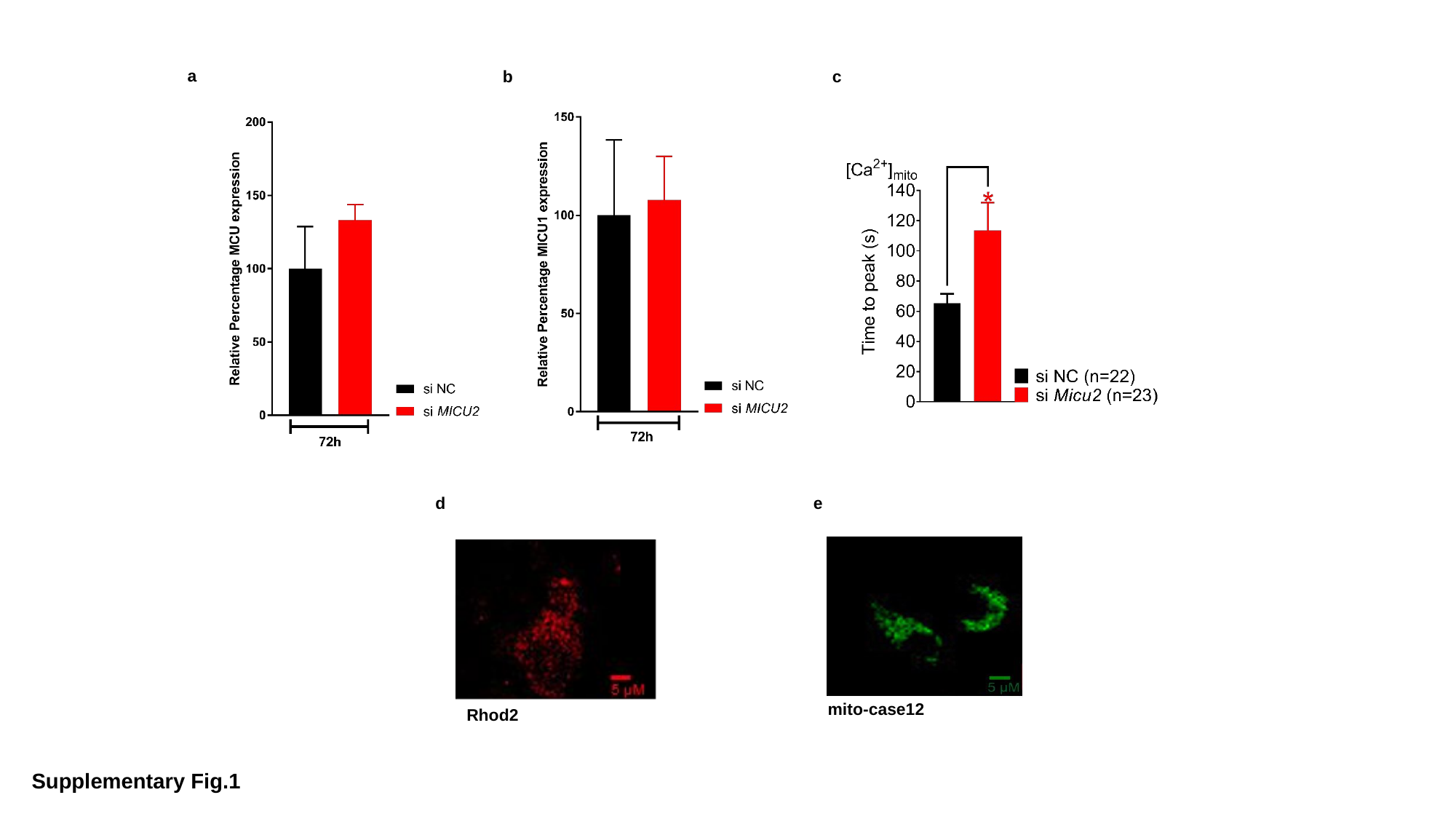

a
b
c
d
e
mito-case12
Rhod2
Supplementary Fig.1

## Slide 2
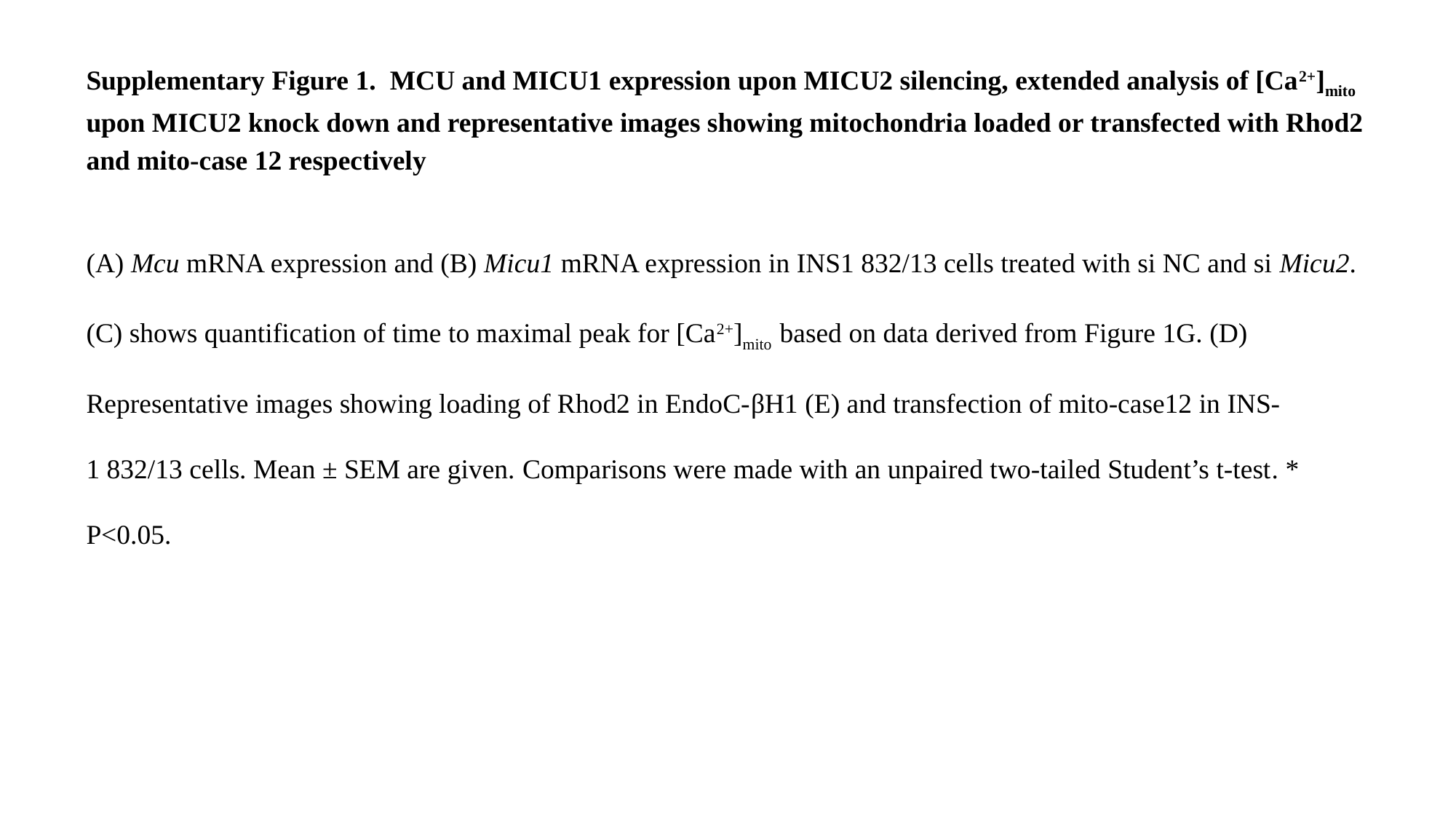

Supplementary Figure 1. MCU and MICU1 expression upon MICU2 silencing, extended analysis of [Ca2+]mito upon MICU2 knock down and representative images showing mitochondria loaded or transfected with Rhod2 and mito-case 12 respectively
(A) Mcu mRNA expression and (B) Micu1 mRNA expression in INS1 832/13 cells treated with si NC and si Micu2. (C) shows quantification of time to maximal peak for [Ca2+]mito based on data derived from Figure 1G. (D) Representative images showing loading of Rhod2 in EndoC-βH1 (E) and transfection of mito-case12 in INS-1 832/13 cells. Mean ± SEM are given. Comparisons were made with an unpaired two-tailed Student’s t-test. * P<0.05.

## Slide 3
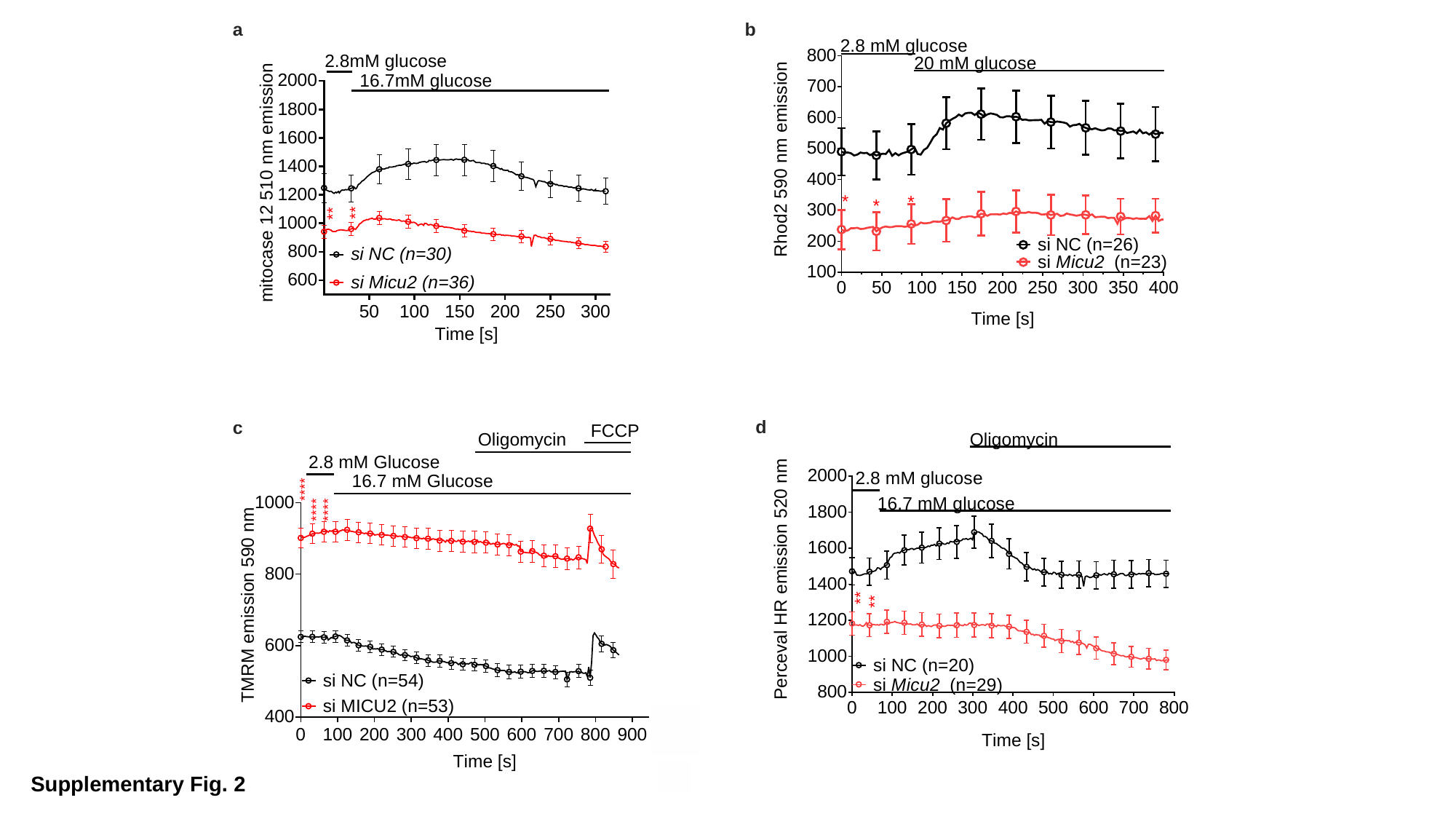

Supplementary Fig. 2

## Slide 4
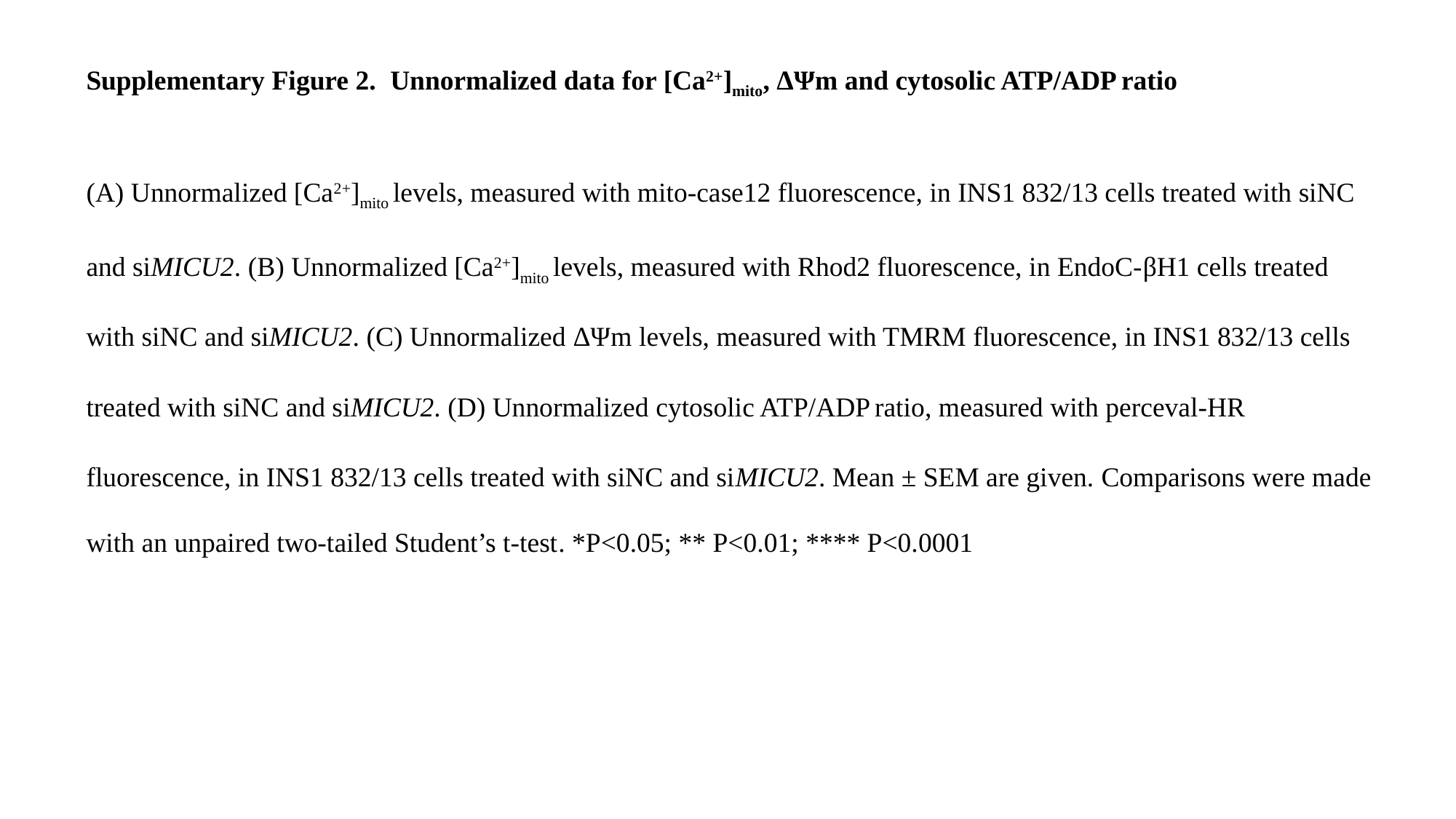

Supplementary Figure 2. Unnormalized data for [Ca2+]mito, ΔΨm and cytosolic ATP/ADP ratio
(A) Unnormalized [Ca2+]mito levels, measured with mito-case12 fluorescence, in INS1 832/13 cells treated with siNC and siMICU2. (B) Unnormalized [Ca2+]mito levels, measured with Rhod2 fluorescence, in EndoC-βH1 cells treated with siNC and siMICU2. (C) Unnormalized ΔΨm levels, measured with TMRM fluorescence, in INS1 832/13 cells treated with siNC and siMICU2. (D) Unnormalized cytosolic ATP/ADP ratio, measured with perceval-HR fluorescence, in INS1 832/13 cells treated with siNC and siMICU2. Mean ± SEM are given. Comparisons were made with an unpaired two-tailed Student’s t-test. *P<0.05; ** P<0.01; **** P<0.0001

## Slide 5
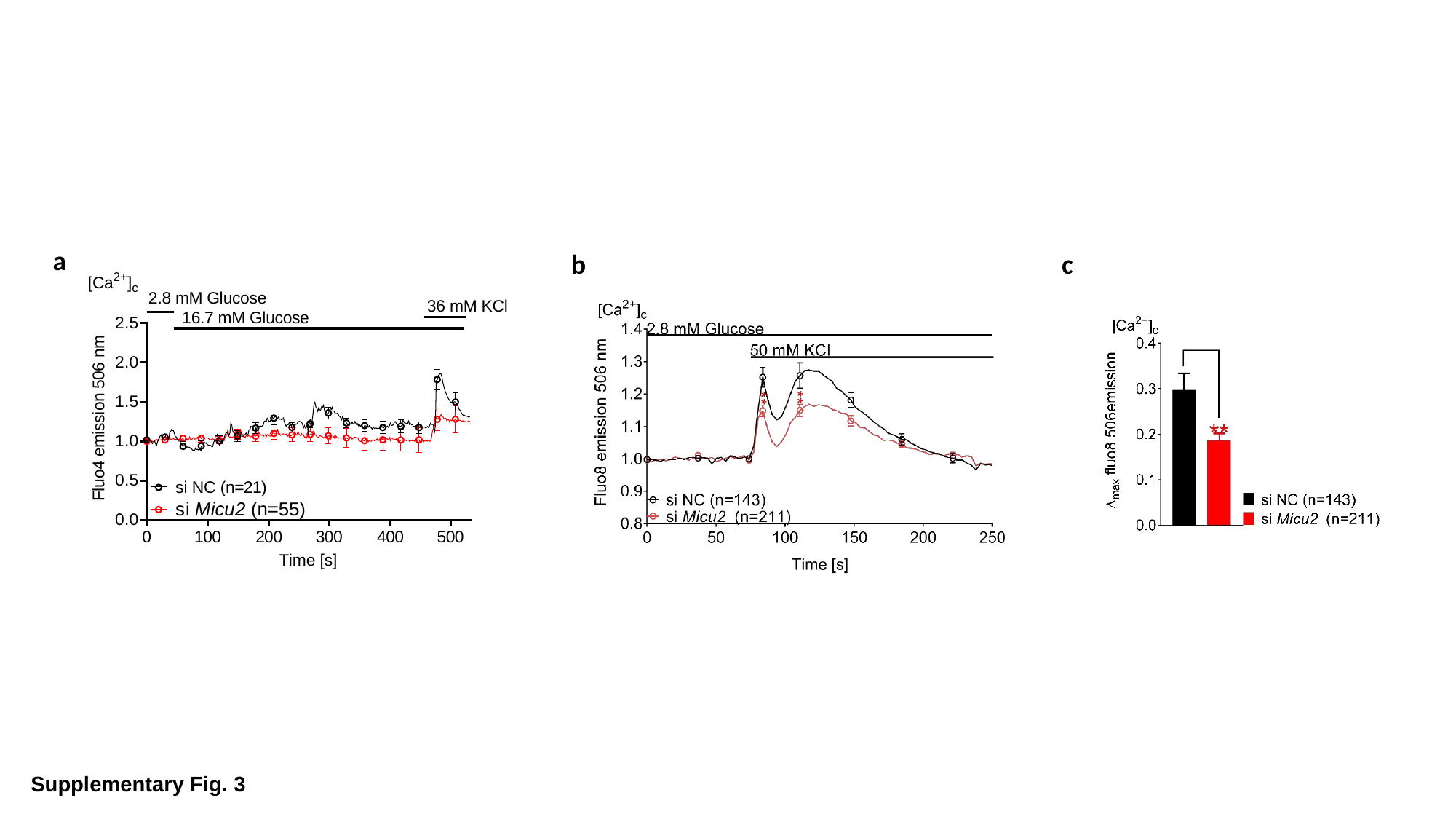

a
b
c
Supplementary Fig. 3

## Slide 6
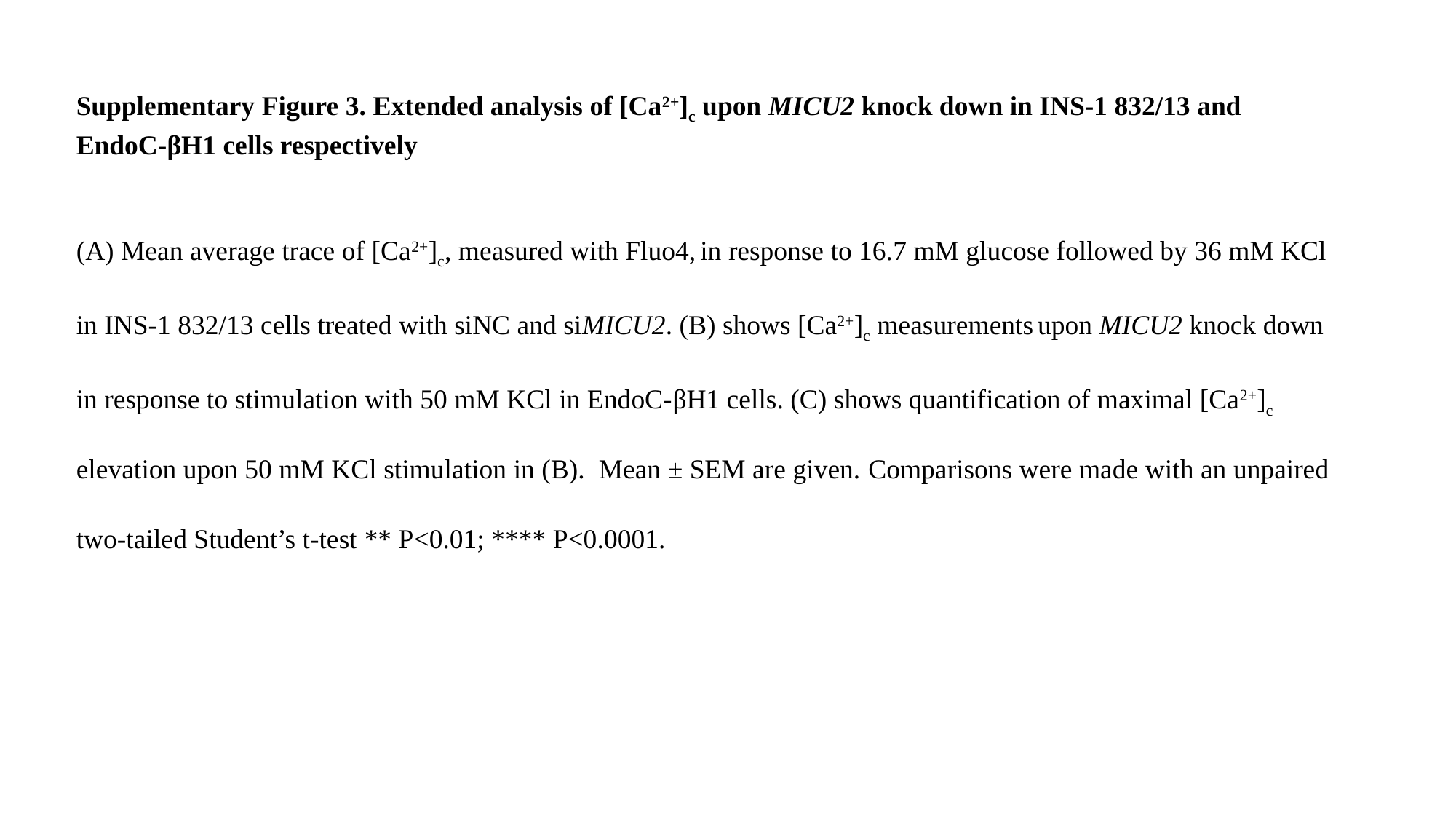

Supplementary Figure 3. Extended analysis of [Ca2+]c upon MICU2 knock down in INS-1 832/13 and EndoC-βH1 cells respectively
(A) Mean average trace of [Ca2+]c, measured with Fluo4, in response to 16.7 mM glucose followed by 36 mM KCl in INS-1 832/13 cells treated with siNC and siMICU2. (B) shows [Ca2+]c measurements upon MICU2 knock down in response to stimulation with 50 mM KCl in EndoC-βH1 cells. (C) shows quantification of maximal [Ca2+]c elevation upon 50 mM KCl stimulation in (B). Mean ± SEM are given. Comparisons were made with an unpaired two-tailed Student’s t-test ** P<0.01; **** P<0.0001.

## Slide 7
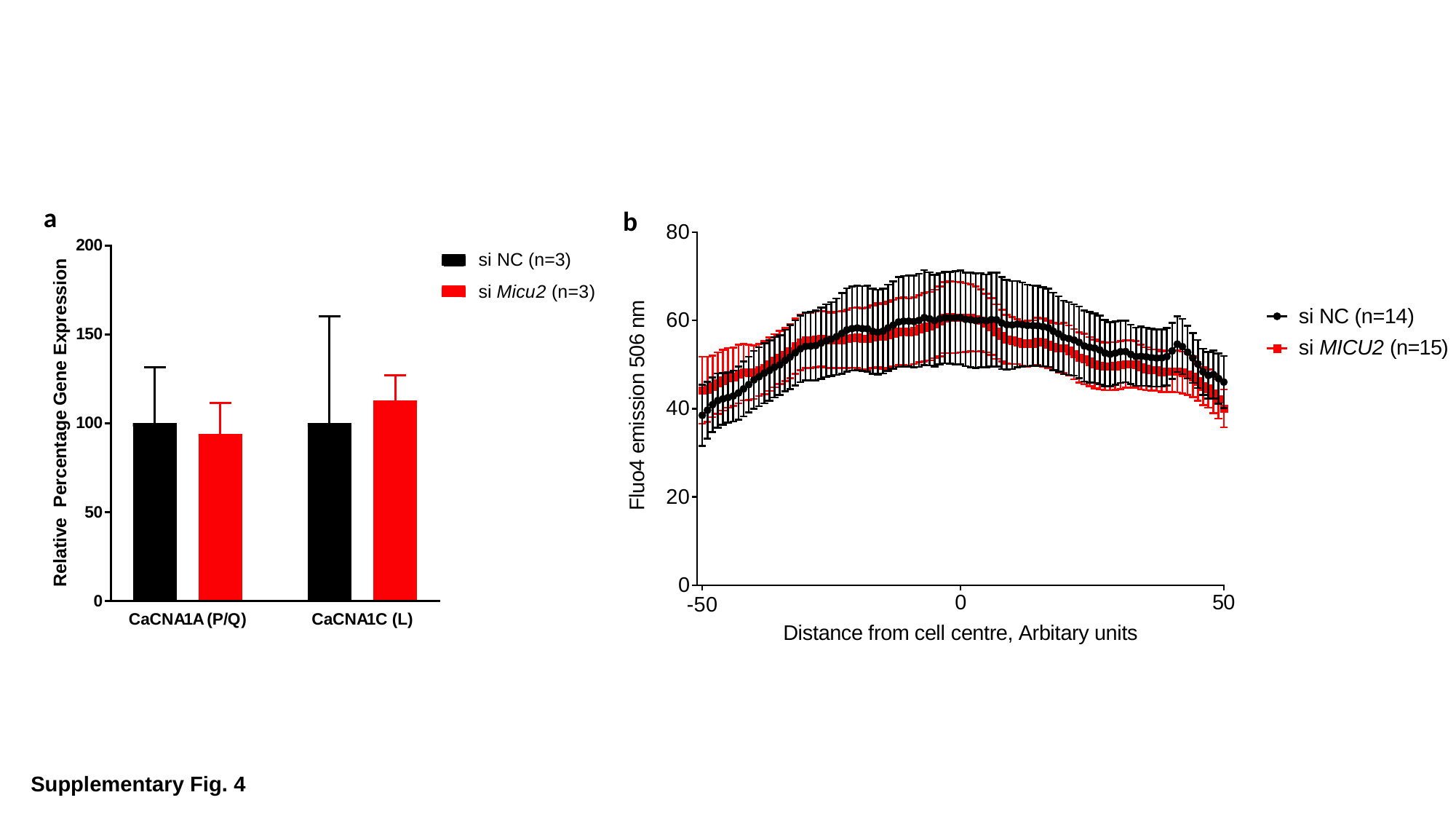

a
b
Supplementary Fig. 4

## Slide 8
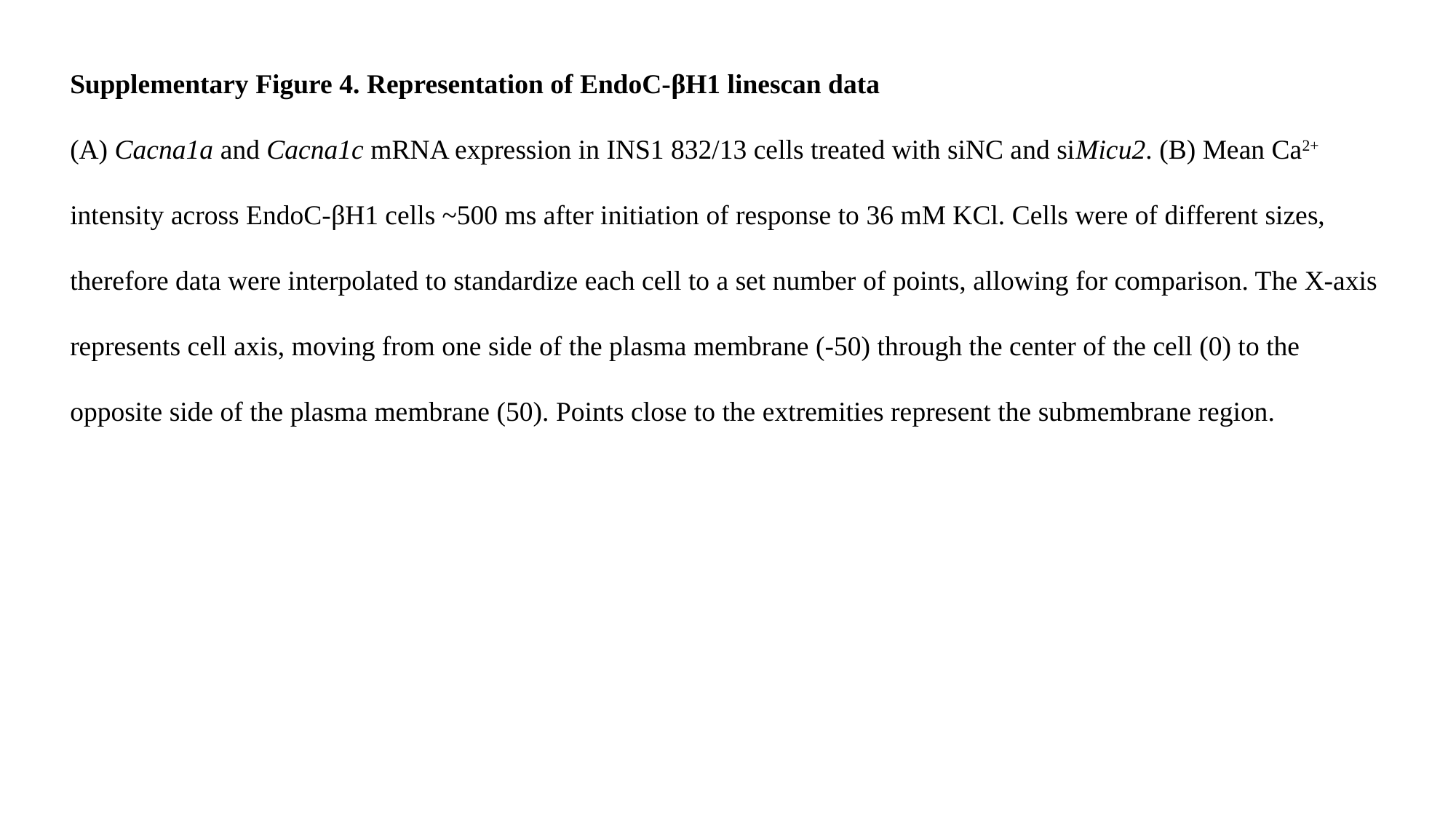

Supplementary Figure 4. Representation of EndoC-βH1 linescan data
(A) Cacna1a and Cacna1c mRNA expression in INS1 832/13 cells treated with siNC and siMicu2. (B) Mean Ca2+ intensity across EndoC-βH1 cells ~500 ms after initiation of response to 36 mM KCl. Cells were of different sizes, therefore data were interpolated to standardize each cell to a set number of points, allowing for comparison. The X-axis represents cell axis, moving from one side of the plasma membrane (-50) through the center of the cell (0) to the opposite side of the plasma membrane (50). Points close to the extremities represent the submembrane region.

## Slide 9
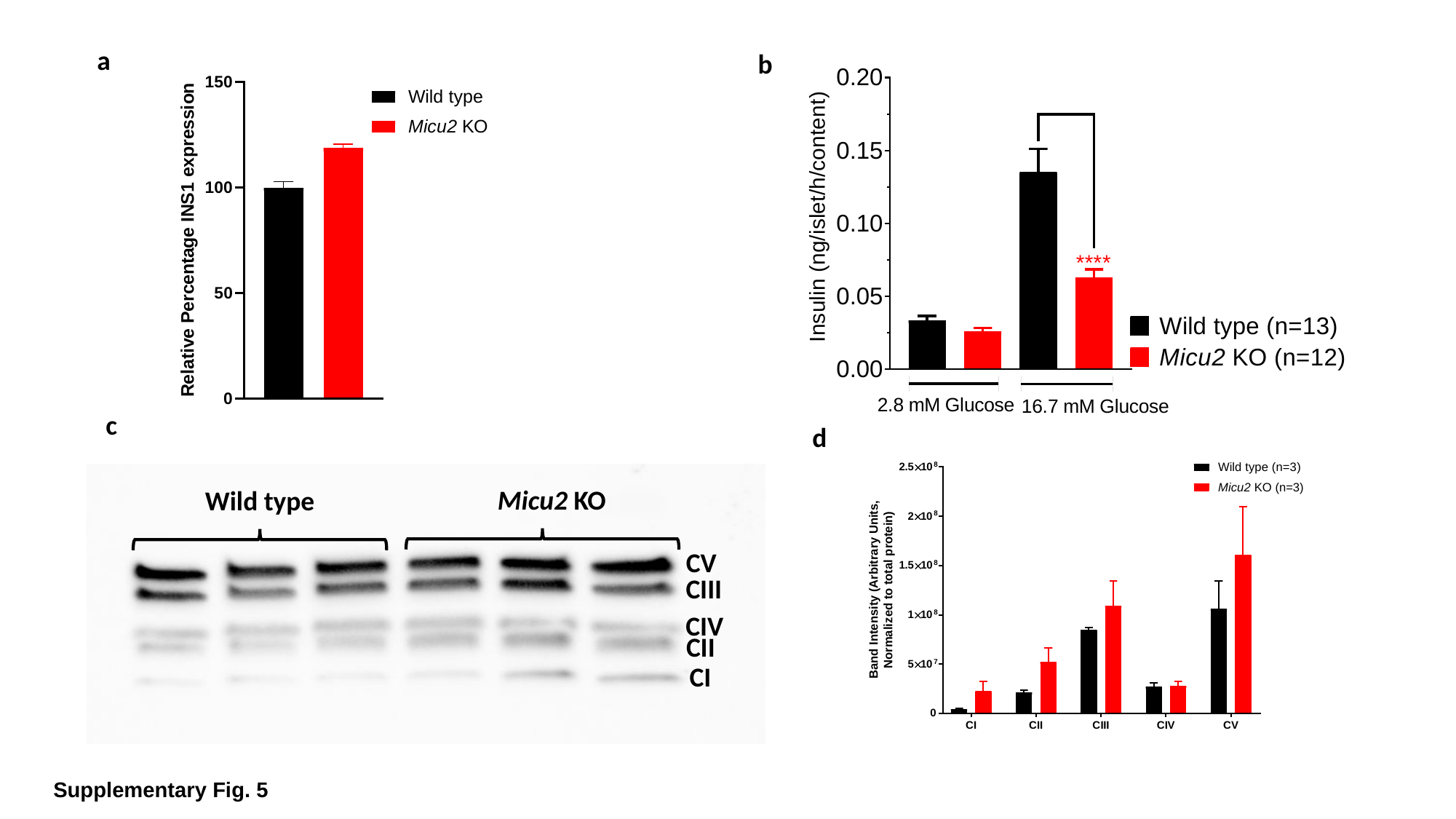

a
b
c
c
d
Wild type
CV
CIII
CIV
CII
CI
Micu2 KO
Supplementary Fig. 5

## Slide 10
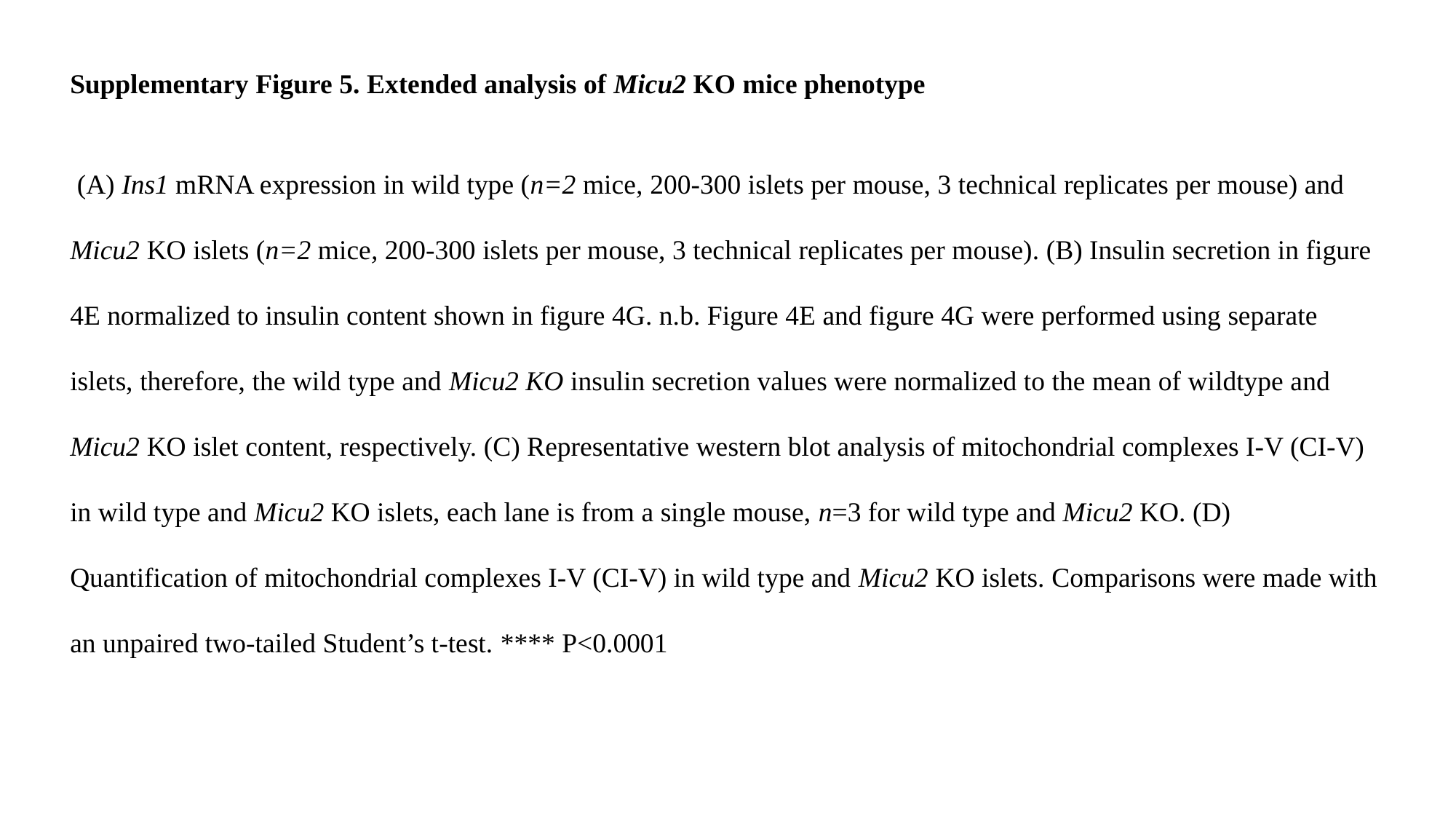

Supplementary Figure 5. Extended analysis of Micu2 KO mice phenotype
 (A) Ins1 mRNA expression in wild type (n=2 mice, 200-300 islets per mouse, 3 technical replicates per mouse) and Micu2 KO islets (n=2 mice, 200-300 islets per mouse, 3 technical replicates per mouse). (B) Insulin secretion in figure 4E normalized to insulin content shown in figure 4G. n.b. Figure 4E and figure 4G were performed using separate islets, therefore, the wild type and Micu2 KO insulin secretion values were normalized to the mean of wildtype and Micu2 KO islet content, respectively. (C) Representative western blot analysis of mitochondrial complexes I-V (CI-V) in wild type and Micu2 KO islets, each lane is from a single mouse, n=3 for wild type and Micu2 KO. (D) Quantification of mitochondrial complexes I-V (CI-V) in wild type and Micu2 KO islets. Comparisons were made with an unpaired two-tailed Student’s t-test. **** P<0.0001

## Slide 11
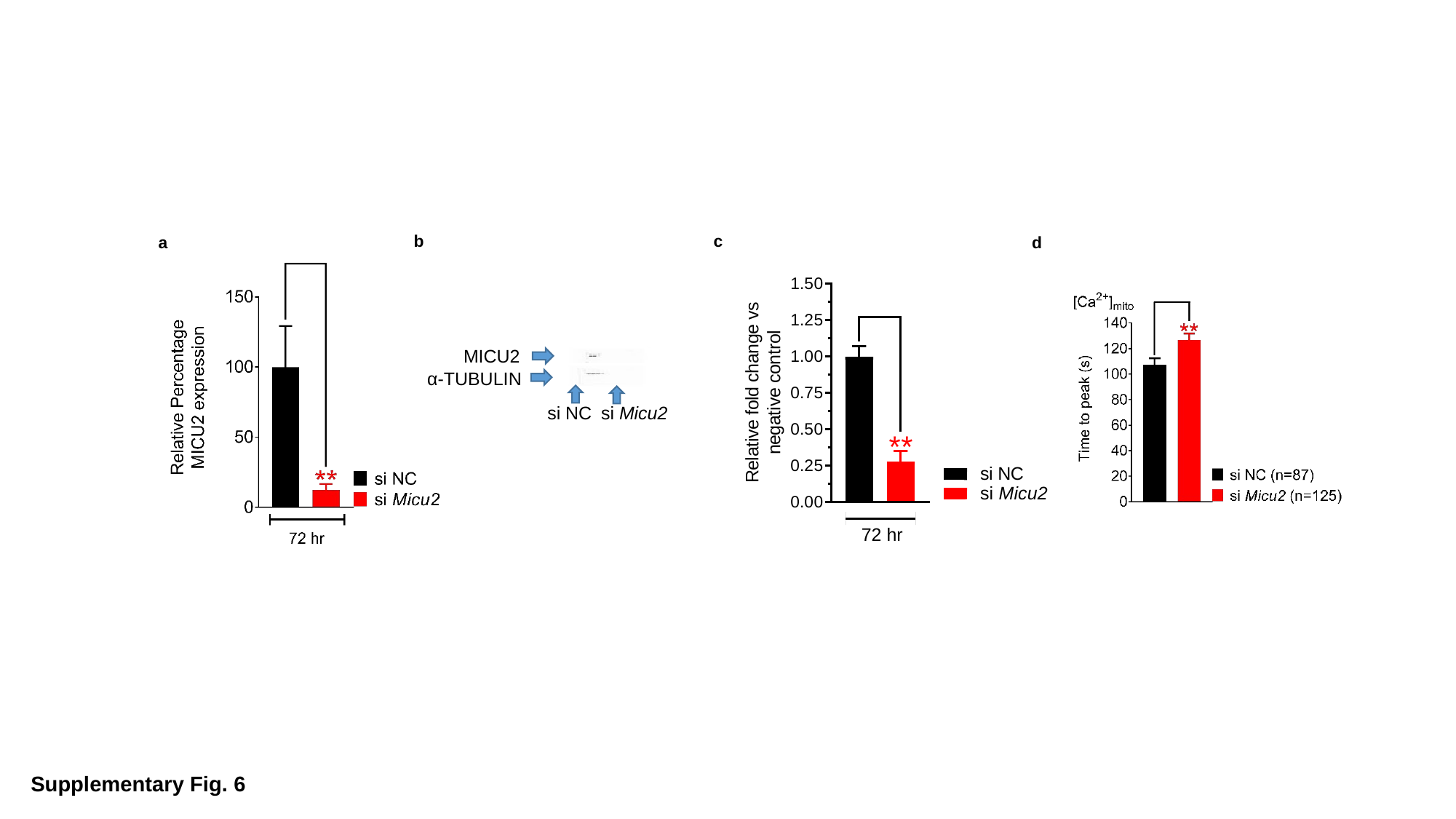

b
c
a
d
MICU2
α-TUBULIN
si NC
si Micu2
Supplementary Fig. 6

## Slide 12
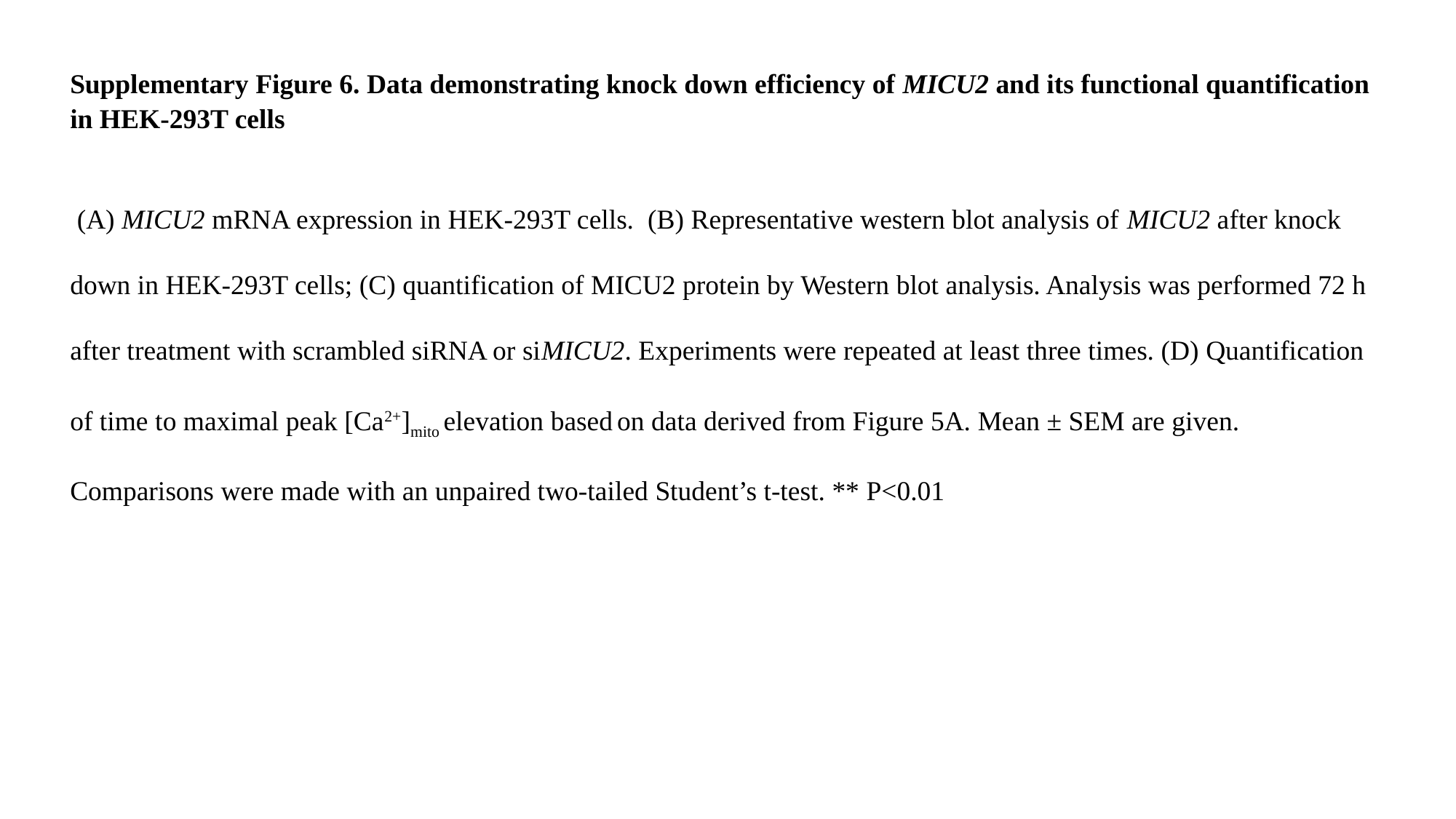

Supplementary Figure 6. Data demonstrating knock down efficiency of MICU2 and its functional quantification in HEK-293T cells
 (A) MICU2 mRNA expression in HEK-293T cells. (B) Representative western blot analysis of MICU2 after knock down in HEK-293T cells; (C) quantification of MICU2 protein by Western blot analysis. Analysis was performed 72 h after treatment with scrambled siRNA or siMICU2. Experiments were repeated at least three times. (D) Quantification of time to maximal peak [Ca2+]mito elevation based on data derived from Figure 5A. Mean ± SEM are given. Comparisons were made with an unpaired two-tailed Student’s t-test. ** P<0.01
